# Cell Invasion Dynamics into a Three Dimensional Extracellular Matrix Fibre Network

**DOI:** 10.1371/journal.pcbi.1004535

**Published:** 2015-10-05

**Authors:** Min-Cheol Kim, Jordan Whisler, Yaron R. Silberberg, Roger D. Kamm, H. Harry Asada

**Affiliations:** 1 BioSystems and Micromechanics IRG, Singapore MIT Alliance for Research and Technology, Singapore; 2 Department of Mechanical Engineering, Massachusetts Institute of Technology, Cambridge, Massachusetts, United States of America; 3 Department of Biological Engineering, Massachusetts Institute of Technology, Cambridge, Massachusetts, United States of America; Cornell University, UNITED STATES

## Abstract

The dynamics of filopodia interacting with the surrounding extracellular matrix (ECM) play a key role in various cell-ECM interactions, but their mechanisms of interaction with the ECM in 3D environment remain poorly understood. Based on first principles, here we construct an individual-based, force-based computational model integrating four modules of 1) filopodia penetration dynamics; 2) intracellular mechanics of cellular and nuclear membranes, contractile actin stress fibers, and focal adhesion dynamics; 3) structural mechanics of ECM fiber networks; and 4) reaction-diffusion mass transfers of seven biochemical concentrations in related with chemotaxis, proteolysis, haptotaxis, and degradation in ECM to predict dynamic behaviors of filopodia that penetrate into a 3D ECM fiber network. The tip of each filopodium crawls along ECM fibers, tugs the surrounding fibers, and contracts or retracts depending on the strength of the binding and the ECM stiffness and pore size. This filopodium-ECM interaction is modeled as a stochastic process based on binding kinetics between integrins along the filopodial shaft and the ligands on the surrounding ECM fibers. This filopodia stochastic model is integrated into migratory dynamics of a whole cell in order to predict the cell invasion into 3D ECM in response to chemotaxis, haptotaxis, and durotaxis cues. Predicted average filopodia speed and that of the cell membrane advance agreed with experiments of 3D HUVEC migration at r^2^ > 0.95 for diverse ECMs with different pore sizes and stiffness.

## Author Summary

Cell invasion into a 3D ECM requires substantial cellular traction forces as well as the degradation of ECM. We are interested in how filopodia gain traction forces from the surrounding collagen fibers in the degradable ECM. Thereby, to create the overall computational model, we integrated four modules, each capturing a different physical aspect influencing migration: 1) filopodia penetration dynamics; 2) intracellular mechanics; 3) reaction-diffusion mass transfer; and 4) structural mechanics of ECM fiber networks. We successfully compared our model with experiments of 3D HUVEC migration for diverse ECMs with different pore sizes and stiffness. Finally, our model reveals the degradation of ECM fiber network plays an important role in filopodia penetration dynamics during both tugging and contractile phases.

## Introduction

Cell migration in the three dimensional extracellular matrix (ECM) plays a crucial role in a wide variety of biophysical processes, such as wound healing, morphogenesis, angiogenesis, tumor growth, and cancer invasion [1,2]. Migration dynamics in 3D are significantly different from those observed on 2D ECM surfaces [3]. Cells invade into the ECM, extend filopodia into the gel, and degrade and remodel the surrounding ECM. Cells sense the direction and magnitude of complex cues and exhibit multifaceted responses, such as chemotaxis, haptotaxis and durotaxis responses from the 3-D extracellular environment.

Filopodia play many important roles in interacting with ECM. Detailed mechanisms of filopodia dynamics have previously been studied for 2D behaviors: filopodia traction dynamics, including motor-clutch mechanism at the filopodial shaft, frictional slippage, and load-and-fail phenomena at hard and soft ECMs [4]; filopodial protrusion due to actin polymerization and depolymerization [5–7]; the formation of focal complexes (FCs) [8,9] and retraction force generation at the filopodial tip [10]; and filopodium buckling instability [11].

In contrast to their behavior on 2D surfaces, filopodia in 3D must penetrate through and interact with the surrounding ECM fibers, probing for an open space or gap in the ECM fibers to extend. When they encounter an ECM fiber, they bind to it via FCs, and subsequently generate traction forces to pull the cell membrane forward. This complex process and the intricate interactions that occur between the filopod and the ECM fibers remain poorly understood. How are these multifaceted activities coordinated to allow first the filopod and eventually the whole cell to penetrate into the 3D ECM? What are the essential features of 2D filopodia dynamics needed to predict 3D migration? To address these questions, we have conducted experiments to probe the detailed interactions as a single filopodium penetrates into a 3D gel matrix, and constructed a computational model to predict coordinated filopodia behaviors and cell-ECM interactions in 3D.

Our experimental observations using 2D and 3D time-lapse data revealed that filopodia a) crawl or slide along ECM fibers near the tip, b) tug on the fibers to generate local forces, causing deformations within the surrounding gel, and c) probe the local fiber network and coordinate their multiple activities: protrusive outgrowth, retraction, and contraction. To explain these behaviors we have built a computational model in which the crawling/sliding of filopodial tips along fibers is described as a continuous process of forming and rupturing focal complexes (FCs) between integrins distributed along the filopodial shaft and ligands on the ECM fibers. The strength of FCs is determined by binding kinetics and other factors, including the local stiffness and porosity of the ECM. Each filopodium alternates between different modes of behavior depending on the properties of FCs. We have established simple rules to describe the switching and coordination of multifaceted filopodia behaviors. The model reproduced not only our experimental data, but also many of the previously reported behaviors, including frictional slippage, and load-and-fail phenomena at hard and soft ECMs [4].

Predicting the filopodia-ECM interactions and the resultant penetration of the whole cell into a 3D gel matrix requires a detailed model of the ECM. We have constructed a network model of cross-linked ECM fibers, with specified pore size and fiber stiffness to match the experimental measurements of bulk elastic moduli. This fiber network consists of straight fibers having both extensional and bending stiffnesses [12,13]. The ECM fiber network degrades due to matrix metalloproteinases (MMPs) [14,15]; the expression of proteolytic enzymes at the cell membrane [16] dissolves the fiber, ruptures the fiber, or ruptures the cross-link [17,18]. Over the ECM network, chemo-attractants diffuse and react to the cell. Furthermore, the mechanics of the cell membrane and nucleus as well as acto-myosin contraction and actin stress fiber formation are all coupled dynamics inside the cell and the filopodium that must be integrated with the filopodial / ECM dynamics.

Previously, many modeling approaches in the areas of cell migration have already provided insights into processes of cell-adhesion and cancer cell invasion. Such approaches include a coarse-grained Langevin dynamics model of lamellipodium protrusion by actin polymerization [19], an invadopodia penetration model with the effect of crosslink on ECM degradation [17], a force-based, individual-based modeling framework that links single cell migration and ECM fibers through contract guidance and matrix remodeling [18], a multi-scale model of dynamic of cell colonies [20], and a cell invasion model in fiber networks and confined microchannels using extended cellular Potts model (CPM) [21].

Our modeling approach exhibits both mechanistic and chemical interactions of 3-D ECM with filopodial and cellular membrane structures since the degradation of ECM is more essential as the cell penetrates into stiffer and denser ECM [22]. Mechanistic interactions of ECM induce local compaction [23], migration and remodeling of individual ECM fibers [24]. On the other hand, chemical interactions of ECM guide dynamic variations of filopodial orientations due to growth factor gradients in the ECM gel as well as the degradation of ECM fibers due to the secretion of focalized proteolysis at the cellular membrane [25]. As further evidence for the model, we also find the densification of ECM fibers in surroundings of the filopodia at both simulation and experiment since filopodia create a substantial traction force and corresponding ECM fibers are considerably deformed. In addition, our modeling approach is developments of previous our modeling works of intracellular mechanics; an integrative cell migration model incorporating FA dynamics, cytoskeleton and nucleus remodeling, actin motor activity, and lamellipodia protrusion was developed for predicting 1) cell migration behaviors on 3D curved surfaces, such as cylindrical lumens in the 3D ECM [26], and 2) cell spreading and migration behaviors on micropatterns and planar substrates with various fibronectin coating concentrations [27].

Integrating these we predicted the average speeds at which the filopodium and cell penetrate into the ECM, obtaining excellent agreement (*r* ^2^ = 0.995) with experiment data for diverse pore sizes and gel moduli. To our knowledge, no cell invasive model that takes into account penetration of the cell into a 3D ECM environment has previously been reported that reflects both the cell-ECM and filopodia-ECM interactions.

## Results

### Cell invasion dynamics model

**Fig 1:**
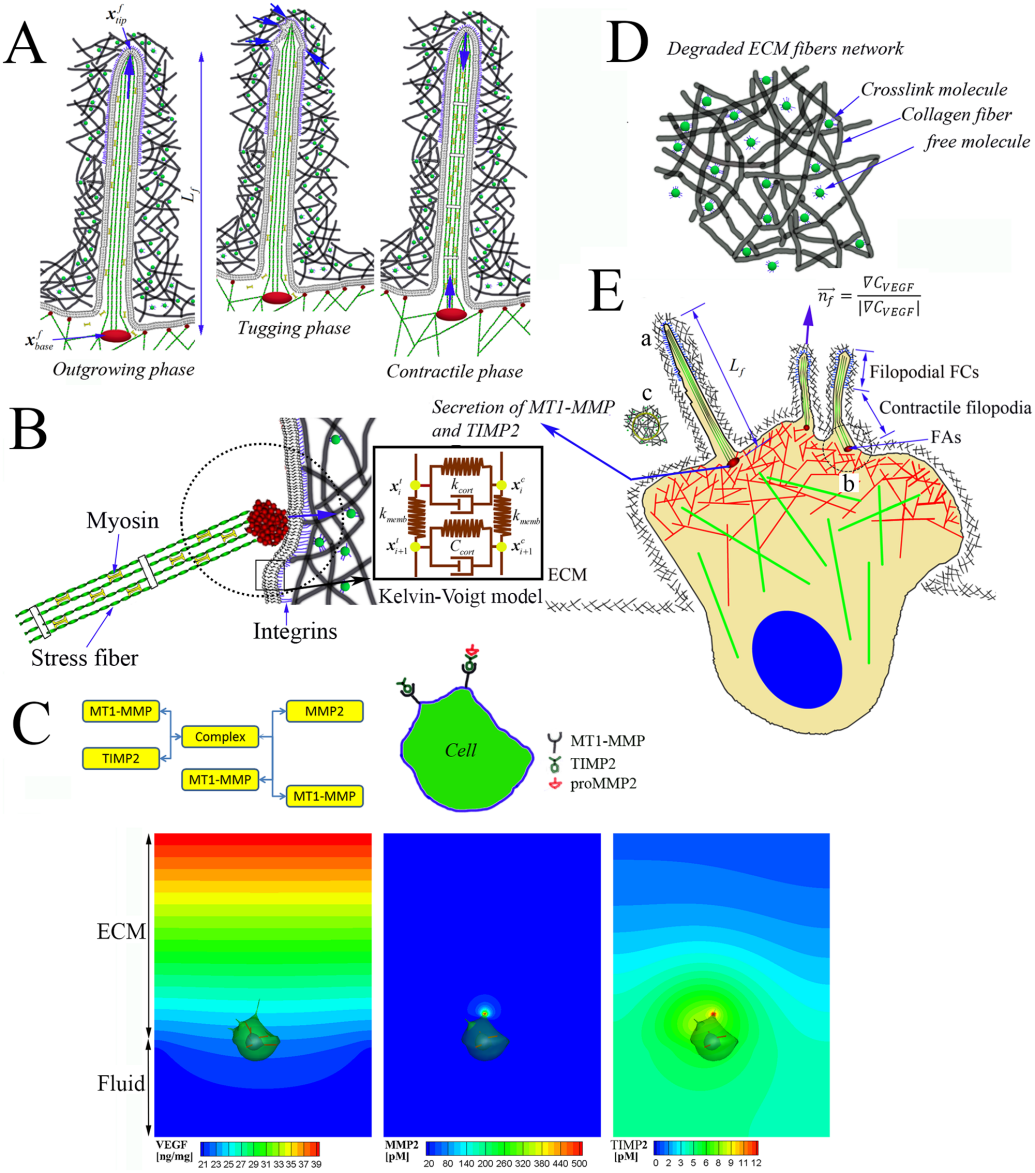
Four modules for the cell invasion dynamics. **A**) Filopodia dynamics showing outgrowing, tugging and contractile phases (see ‘a’ in **E**). **B**) cellular membrane mechanics showing the membrane is not only connected by actin stress fibers, but also anchored to elastic ECM fibers by forming focal adhesions (FAs) (See ‘b’ in **e**), and viscoelastic behaviors in cellular membrane is modeled using Kelvin-Voigt model. **C**) Schematic diagram of MMP-2 activation [14] and selected simulation results of VEGF, MMP-2 and TIMP-2 concentration distributions using simplified model for MMP-2 activation. **D**) a magnified schematic of ‘c’ in **E)** showing the elastic ECM fiber networks is organized with collagen fibers, crosslinking molecules and free (or non-crosslinked) molecules. **E**) An integrated schematic representation of MT1-MMP and TIMP-2 secretions at the membrane near the roots of filopodia; directions of outgrowing filopodia are guided when tips of filopodia sense local gradients of VEGF toward a chemotactic cue.

**Fig 2:**
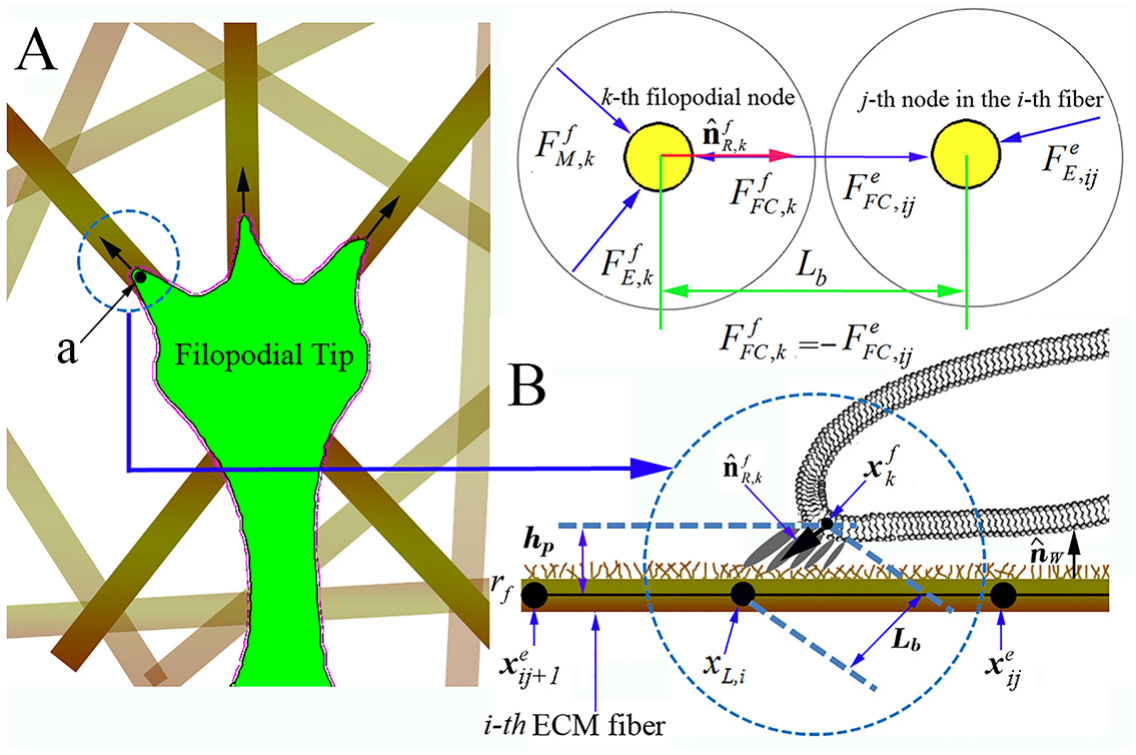
Stochastic model of filopodia penetration dynamics in 3-D ECM fiber network. A) Schematic representation of three focal complexes (FCs) (‘a’) moving along ECM fibers in different directions. Free body diagram of the *k*-th filopodial node and the *j*-th node in the *i*-th fiber in the circle marked in **A)**, where three and two external forces are acting, respectively. Note that, the sum of $F_{FC,k}^{f}$ and $F_{FC,f,ij}^{e}$ is zero. **B**) a magnified view in **A)**, showing the structure of FC including an integrin node at the filopodial membrane to an underlying ECM fiber, illustrating a stochastic ligand-receptor bonding process at the FC site. Note that, **A**) and **B**) represent top and side views, respectively.

**Fig 3:**
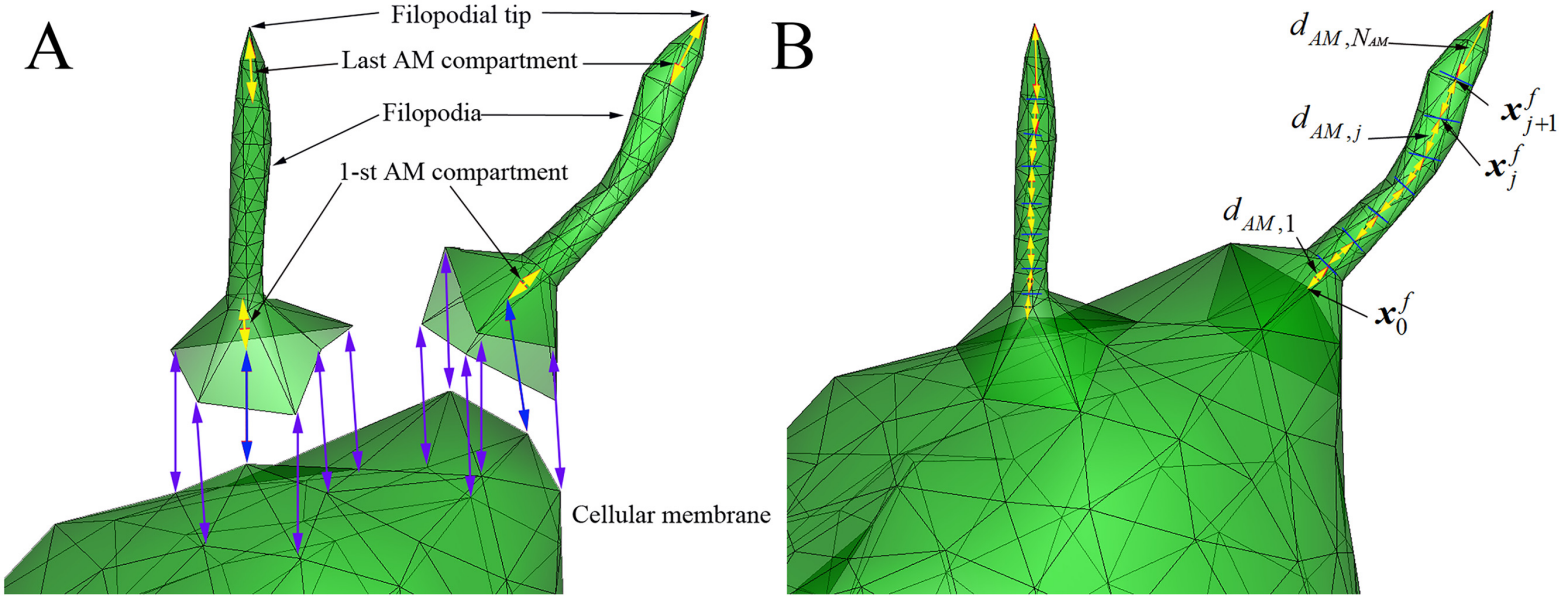
Geometric structure of filopodia model. **A**) Roots of filopodia are attached to the cellular membrane (six blue arrows), and the 1^st^ and last AM (acto-myosin) compartments are attached to the cellular membrane and the filopodial tip, respectively (yellow arrows). **B**) The filopodia in A) attached to the cellular membrane model: yellow arrows represent eight AM compartments in each filopodium.

To create the overall computational model, we integrate four modules, each capturing a different physical aspect influencing migration: 1) filopodia penetration dynamics [4] (Fig 1A); 2) intracellular mechanics, including formation of FAs and actin stress fibers (SFs), and remodelling of cellular and nuclear membranes [26,27] (Fig 1B); 3) reaction-diffusion mass transfer [14,15] (Fig 1C); and 4) dynamics of ECM fiber networks [12,13] (Fig 1D). In particular, it should be noted that FAs are different from FCs in that FAs (1–5 μm in size) are formed on the cellular membrane with a long turnover time > 5 minutes, but FCs (~0.5 μm in size) are formed on the filopodial membrane with a short turnover time < 5 minutes [28]. To incorporate viscoelastic behaviors in cellular membrane, line elements of actin cortex in the cellular membrane can be modeled using Kelvin-Voigt model (a spring and a dashpot together in parallel) (Fig 1B). The detailed equations that govern each of these dynamical processes are summarised in Table 1, and the list of simulation parameters are also summarised in Table 2.

**Table 1:** Dynamical models.

Module	Equation	Numerical method	Couplings
F	$C_{f}\;\frac{dx_{i}^{f}}{dt}=F_{E,i}^{f}+F_{FC,i}^{f}+F_{P,i}^{f}+F_{AM,i}^{f}\;,\;i=1,\cdots ,N_{f}.$	RM	CC, E and RD
E	$C_{e}\;\frac{dx_{i}^{e}}{dt}=F_{FA,i}^{e}+F_{FC,i}^{e}+F_{E,i}^{e}\;,\;i=1,\cdots ,N_{e}.$	RM	F, CC and RD
CC	$\left(C_{c}+C_{cort}\right)\frac{dx_{i}^{c}}{dt}-C_{cort}\frac{dx_{i}^{t}}{dt}=F_{FA,i}^{c}+F_{E,i}^{c}+F_{L,i}^{c}+F_{T,i}^{c},\;i=1,\cdots ,N_{c}$	RM	F, E, CT and RD
CT	$-C_{cort}\frac{dx_{i}^{c}}{dt}+\left(C_{t}+C_{cort}\;\right)\frac{dx_{i}^{t}}{dt}=F_{E,i}^{t}+F_{SF,i}^{t}+F_{T,i}^{t},\;i=1,\cdots ,N_{t}$	RM	CC and CN
CN	$C_{n}\frac{dx_{i}^{n}}{dt}=F_{E,i}^{n}+F_{SF,i}^{n},\;i=1,\cdots ,N_{n}$	RM	CT
RD	$\frac{\partial C_{X_{1i}}}{\partial t}=\nabla \cdot \left(D_{X_{1i}}\nabla C_{X_{1i}}\right)+R_{X_{1i}}$	FVM	CC,F and E
	$\frac{\partial C_{X_{2i}}}{\partial t}=R_{X_{2i}}$	FVM	CC

The four modules describe the dynamics of the filopodia (F), cell (C) and ECM (E) modules and the Reaction-diffusion (RD) modules. F module: $F_{E,i}^{f}$, $F_{FC,i}^{f}$,$F_{P,i}^{f}$ and $F_{AM,i}^{f}$ are the elastic force, focal complex force, force due to protrusive polymerization of actin filaments; and contractile force due to acto-myosin (AM) motor activity at the *i*-th filopodial node, respectively. C module: C is composed of three sub-modules representing the cellular membrane (CC), the transduce layer (CT) and the nuclear membrane dynamics (CN). CC module:$F_{FA,i}^{c}$, $F_{E,i}^{c}$, $F_{L,i}^{c}$, and $F_{T,i}^{c}$ are the focal adhesion force, the elastic energy force, lamellipodium force, and cortical tension force at the *i*-th cell membrane node, respectively. CT:$F_{E,i}^{t}$,$F_{SF,i}^{t}$, and $F_{T,i}^{t}$ are the elastic force, SF force, and cortical tension force at the *i*-th force transduce node, respectively. CN: $F_{E,i}^{n}$ and $F_{SF,i}^{n}$ are the elastic force and SF force at the *i*-th nuclear membrane node, respectively. E: $F_{FA,i}^{e}$,$F_{FC,i}^{e}$ and $F_{E,i}^{e}$ are the FA force, FC force and elastic force at the *i*-th ECM fiber node, respectively. Here, *C*_*f*_, *C*_*c*_, *C*_*t*_, *C*_*n*_ and *C*_*e*_ are friction coefficients associated with the energy dissipation at F, CC, CT, CN and E modules, respectively. In addition, *C*_*cort*_ is a drag coefficient associated with viscoelastic behaviours in the actin cortex. All dynamic models of simulations are carried out using a fourth order Rosenbrock method based on an adaptive time-stepping technique for integrating ordinary differential equations with convergence criterion < 10^−4^. F module is geometrically coupled with CC module and with E module through, $F_{FC,i}^{f}=-F_{FC,j}^{e}$. CC module is coupled with E module with equations, $F_{FA,i}^{c}+F_{FA,j}^{e}=0$. CC module is coupled with CT module with equations, $F_{T,i}^{c}+F_{T,i}^{t}=0$. CT module is coupled with both CN modules with an equation, $F_{SF,i}^{t}+F_{SF,j}^{n}=0$. RD module is divided into two subgroups according to whether or not the diffusion terms are included (See also Table 2). RD with diffusion: *X*_1_ is the concentration of VEGF, MMP-2 and TIMP-2, or ligand at both the ECM and medium domains. Here, medium domain means the microfluidic channel or blood vessel, and the cell adhere to the interface between ECM and medium domains (See Fig 1C). *D*_*VEGF*_, *D*_MMP2_, *D*_TIMP2_ and *D*_*Ligand*_ are the diffusion coefficients of VEGF (68.8×10^−12^ m^2^/s), MMP-2 (68.8×10^−12^ m^2^/s), TIMP-2 (1.29×10^−12^ m^2^s^-1^) and ligands (1.0×10^−15^ m^2^s^-1^), respectively. RD without diffusion: *X*_2_ is a biochemical concentration of MT1-MMP, or complex of TIMP2-MT1-MMP-MMP2 at the cellular membrane domain. All of RD modules are solved using Finite Volume Method (FVM) with convergence criterion < 10^−3^.

**Table 2:** List of simulation parameters.

Parameter	Definition	Value	Sources
*A*	Area [μm^2^]		
*A*_*AM*_	Averaged AMs' cross-sectional area in a filopodium [μm^2^]	7.07×10^−2^	[5]
*A*_*f*_	Averaged cross-sectional area of a single fiber [μm^2^]	(0.615~1.32)×10^−3^	
*C*_*c*_	Friction coefficients associated with the energy dissipation at the integrin node [N s m^-1^]	0.001	C
*C*_*cort*_	Drag coefficients associated with viscoelastic behaviors in actin cortex	0.006	C
*C*_*e*_	Friction coefficients associated with the energy dissipation at the ECM fiber node [N s m^-1^]	0.001	C
*C*_*f*_	Friction coefficients associated with the energy dissipation at the filopodial node [N s m^-1^]	0.001	C
*C*_*t*_	Friction coefficients associated with the energy dissipation at the transduce node [N s m^-1^]	0.001	C
*C*_*n*_	Friction coefficients associated with the energy dissipation at the nuclear node [N s m^-1^]	0.001	C
*F*	Force [N]		
***$F_{P,\text{max}}^{f}$***	Maximum value of the force due to actin polymerization [nN]	2	C
*E*_*AM*_	Young's modulus value of AMs [kPa]	230	[37]
***$E_{f}^{e}$***	Young’s modulus value of single fiber [MPa]	1	C
*H*_*AM*_	Total elastic energy stored in the AMs in the filopodium [pJ]		
*L*	Length		
*L*_*b*_	Stretched length of bonds between receptors and ligands		
$L_{AM,i}^{1}$	Length of the *i*-th single unit of AMs at the present time [nm]		
$L_{AM,i}^{0}$	Length of the *i*-th single unit of AMs at the previous time [nm]		
$L_{i\;j}^{e}$	Stressed length of the *j*-th segment of the *i*-th fiber [μm]		
$L_{i\;j}^{e0}$	Unstressed length of the *j*-th segment of the *i*-th fiber [μm]		
*N*_*f*_	Number of nodes at filopodial membrane	60~180	
*N*_*e*_	Number of nodes at ECM fiber networks	30k~234k	
*N*_*c*_	Number of nodes at cellular membrane	549	
*N*_*t*_	Number of nodes at transduce layer	549	
*N*_*n*_	Number of nodes at nuclear membrane	549	
***$N_{i}^{e}$***	Number of nodes at the i-th fiber		
*N*_*AM*_	Number of contractile compartments of AM assemblies		
*d*_*AM*,*j*_	Distance of the *j*-th contractile compartment of AM assemblies [nm]		
*h*_*p*_	Height from the surface to the *i*-th integrin node [nm]		
*κ*_*cort*_	Effective spring constant of line elements of the actin cortex [N/m]	8×10^−3^	C
*κ*_*LR*_	Effective spring constant of ligand-receptor bond [pN/nm]	1.0	[33]
*κ*_*AM*,*j*_	Effective spring constant of the j-th AM assemblies in the filopodium [pN/nm]	20.32~33.87	C
***$\kappa_{f,s}^{e}$***	The stretching modulus of a fiber [nN]	0.615~1.32	C
***$\kappa_{f,b}^{e}$***	The bending modulus of a fiber [pN μm^2^]	(3.02~12.81)×10^−3^	C
*κ*_*memb*_	Effective spring constant of line elements of the cell membrane [N/m]	5.0×10^−5^	[37]
***$\theta_{i\;j}^{e}$***	Stressed angle at the *j*-th node between two segments in the *i*-th fiber		
***$\theta_{i\;j}^{e0}$***	Unstressed angle at the *j*-th node between two segments in the *i*-th fiber		
*k*_*off*_	Kinetic dissociation rate [s^-1^]		
$k_{off}^{0}$	Kinetic dissociation rate at an unstressed state [s^-1^]	1	C
***$n_{b}^{k}$***	Number of bonds between integrins and ligands at the k-th filopodial node		
***${\hat{n}}_{R,k}^{f}$***	Unit vector normal to the local surface of the *k*-th filopodial node		
${\hat{n}}_{VEGF,i}^{f}$	unit normal vector parallel to the gradient of *C* *_VEGF_* at the i-th filopodial node		
***${\hat{n}}_{w}$***	Unit normal vector at the local surface of the fiber		
*t*	Time [s]		
***${\hat{t}}_{i,k}$***	Tangential unit vector at the *k*-th segment in the *i*-th fiber		
***v***	Velocity vector [nm/s]		
*v*_*m*_	Sliding rate of non-muscle myosin II on the actin filaments [nm/s]		[42]
*v*_*m*0_	Sliding rate of non-muscle myosin II in the absence of load [nm/s]		
***x***	Location vector [μm]		
*x*_*L*,*i*_	Root of ligand-receptor bonds on the local surface of a fiber [nm]		
$x_{ij}^{e}$	The *j*-th location vector along to the *i*-th fiber [μm]		
***λ***	Equilibrium distance of an integrin [nm]	30	[34]
**Sup**			
*c*	cytoskeleton		
*e*	extracellular matrix		
*f*	filopodia		
*n*	nucleus		
*i*	*i*-th node		
*t*	transduce layer		
*0*	Previous time or initial state		
*1*	Present time		
**Sub**			
*AM*	Acto-myosin		
*E*	Elastic		
*FA*	Focal adhesion		
*FC*	Focal complex		
*P*	Actin polymerization		
*SF*	Stress fiber		
*T*	Transduce layer		
*b*	bonds		
*c*	cytoskeleton		
*e*	extracellular matrix		
*f*	filopodia		
*n*	nucleus		
*t*	transduce layer		

*C means “current work”.

### Filopodia penetration dynamics

We identified and modelled four phases of filopodial dynamics. Three resemble the phenomena previously reported: an outgrowing phase due to protrusive polymerization of actin [5–7]; a retractile phase due to zero or weak FC forces at the filopodial tip and fast myosin motor activities along the filopodial shaft [9]; and a contractile phase due to strong FC forces at the filopodial tip and slow myosin motor activities along the filopodial shaft [4,9] (Fig 1A). The fourth phase, which we refer to as the “tugging phase”, begins with the formation of FCs near the tip of the filopodium. The point of attachment between a filopodial tip and a nearby ECM fiber migrates along the fiber, a tension builds up between them as the filopodium moves, and the bond either ruptures or results in the generation of a significant traction force, depending on the strength and spatiotemporal properties of the FC formation. This phase plays a critical role in switching among the other phases and coordination of the diverse filopodial dynamics, leading to either success or failure of cell migration depending on local ECM conditions.

First, in the outgrowing phase, polymerization of actin filaments generates a force $F_{P,i}^{f}$ on the filopodial membrane [29]. The average magnitude of the maximum protrusive actin pressure is in the range of 5–10 nN/μm^2^. It has been reported that a few nN of force can exerted by a few hundred actin filaments per μm^2^, which implies that each filament contributes on the order of 10–20 pN [30]. Thereby, the imposed maximum magnitude of $F_{P,i}^{f}$~ 2 nN ($F_{P,\text{max}}^{f}$) is reasonable since the diameter of single filopodium is assumed to be 300 nm consisting of >30 actin filaments. It should be noted that $F_{P,i}^{f}$ is exerted only at the tip of the filopodium. To incorporate a chemotactic response from the 3D ECM environment, the direction of $F_{P,i}^{f}$ is predicted from the gradient of the VEGF concentration (*C*_*VEGF*_) at the tip of the filopodium. Thus, $F_{P,i}^{f}$ is given by $F_{P,i}^{f}=F_{P,\text{max}}^{f}{\hat{n}}_{VEGF,i}^{f}$ where ${\hat{n}}_{VEGF,i}^{f}$ is a unit normal vector parallel to the gradient of *C*_*VEGF*_ (Fig 1E).

Second, the contractile phase arises from the acto-myosin contraction. At contractile phase, we assume that a bundle of actin microfilaments is assembled by actin-myosin II interactions [31]. It has been observed that veil (or lamellae) advances following filopodial pulling (or contractile) motion, a process in which two kinds of adhesion mechanisms with collagen fibers in the ECM take place, i.e., FCs formation at the tip of filopodial shaft and FAs formation at the root of filopodium [32]. These two kinds of adhesions at both tip and root of the filopodium are required for gaining stable traction forces from the surrounding collagen fibers in the ECM, and for transmission of the force to allow the advancement of the cellular membrane via the filopodial contractile motion. Here, stable traction forces indicate sufficiently large magnitude to withstand the acto-myosin contractile force. In addition, for the condition of the veil advancement, the focal complex force at the filopodial tip must be stronger than the focal adhesion force at the veil. Otherwise, weak complex force leads to the retractile phase. Therefore, we model filopodia penetration dynamics at the contractile phase by taking into account the complex myosin motor activity and FCs formation at the tip of filopodial shaft.

Third, the retractile phase is also arises from the acto-myosin contraction in a similar manner of the contractile phase. However, the retractile phase is induced by weak traction forces from the surrounding ECM, which result in fast, oscillatory ‘load-and-fail’ traction dynamics during the retractile phase (S1 Fig and S1 Video). That is, the reconnection of FCs at the tip allows a filopodium to repeatedly probe the surrounding ECM fibers, but the filopodium retracts immediately if it experiences weak traction forces at the tip [10].

Finally, we have found through experiments that there exists another phase in filopodial dynamics: the tugging phase (S2 Video). The tip of a filopodium apparently crawls or slides along a nearby ECM fiber or multiple fibers (Fig 2A). We have observed that the binding site of FCs moves along the ECM fibers and that the bound ECM fibers are pushed or pulled by the filopodial tip. The adhesive force of FCs is sufficient to prevent the filopodium from retracting. This crawling or sliding can be viewed as a continuous process during which FCs form and rupture as they move along the ECM fibers, as depicted in Fig 2B.

### Filopodia focal complex formation

Here, we build a computational model to predict the continuous formation and rupture of FCs along the ECM fibers based on binding kinetics between integrins on the filopodial membrane and collagen molecules in ECM fibers. This technique is similar to the one used to predict the formation of a FA at a cellular membrane that interacts with ligands in the ECM fibers [27]. The FC force at the *k*-th filopodial node is given by

$$F_{FC,k}^{f}=n_{{}_{b}}^{k}\;\kappa_{LR}\left(L_{b}-\lambda \right){\hat{n}}_{R,k}^{f}$$ (1)

where $n_{{}_{b}}^{k}$ is the number of integrin-collagen bonds, *κ*_*LR*_ is the spring constant of a single ligand-receptor bond (~1 pN/nm) [33], *L*_*b*_ is the average stretched length of the ligand-receptor bonds, *λ* is an unstressed length of bonds (~30nm) [34] and ${\hat{n}}_{R,k}^{f}$ is a unit vector at the local surface of the *k*-th filopodial node toward the bonding site between the *j*-th and *j+1*-th nodes in the *i*-th fiber (Fig 2B). (*L*_*b*_-*λ*) represents the stretched distance from the equilibrium. From Fig 2B, this intersection position, that is, the root location of receptor and ligand bonds (*x_L,i_*) between $x_{ij}^{e}$ and $x_{ij+1}^{e}$, is given by

$$x_{L,i}=x_{k}^{f}+L_{b}{\hat{n}}_{R,k}^{f}=x_{k}^{f}-\frac{h_{p}{\hat{n}}_{R,k}^{f}}{{\hat{n}}_{w}\cdot {\hat{n}}_{R,k}^{f}}$$ (2)

where *h*_*p*_ is the gap between the *k*-th filopodial membrane node and ECM fiber node, and ${\hat{n}}_{w}$ is a vector normal to the curved surface of the cylindrical fiber, defined by $\left(x_{i\;j+1}^{e}-x_{i\;j}^{e}\right)\times \left(\left(x_{k}^{f}-x_{i\;j}^{e}\right)\times \left(x_{i\;j+1}^{e}-x_{i\;j}^{e}\right)\right)$ and shown in Fig 2B. We utilize Bell’s model to simulate the stochastic nature of bond rupture and formation. Bell’s equation for the kinetic dissociation rate is defined by $k_{off}=k_{off}^{0}\text{exp}\left[\frac{\kappa_{LR}\left(L_{b}-\lambda \right)x_{b}}{k_{b}T}\right]$, where $k_{off}^{0}$ is the kinetic dissociation rate (1 s^-1^) under unstressed condition with an equilibrium distance *λ*, *x*_*b*_ is the separation distance between the bound state and the transition state (0.02 nm), *k*_*b*_ is the Boltzmann constant, and *T* is the absolute temperature [35]. In addition, kbTxb represents the maximum rupturing force exerted on single molecule of ligand-receptor bond (~200 pN).

During the dynamic process, the filopodium switches between these four phases. Depending on the strength of the FC force, the length of the filopodium, the duration of the current phase, and other factors, transitions between phases are determined. This can be modelled as a discrete state transition network detailed in S2 Fig. Briefly, the outgrowing phase switches to the tugging phase when FCs are formed within a specified time, otherwise it switches to the retraction phase. In addition, the formation of FCs is assumed to occur when *h*_*p*_ is less than 100 nm, and is described by a stochastic process due to binding kinetics between receptors and ligands on the surface of ECM fiber. Monte Carlo simulation methods have been established for various ligand-receptor binding kinetics in the literature [27]. Each focal FC consists of a bundle of ligand-receptor bonds (Fig 2B), each of which ruptures and binds stochastically. Let *P*_*b*_ be the probability with which a single receptor binds to a ligand on the ECM fiber during a time interval Δt.

$$p_{b}=1-\text{exp}\left(-k_{on}\Delta t\right)$$ (3)

$$k_{on}=k_{f}A_{L}\left(C_{L}-C_{b}\right)$$ (4)

where *k*_*f*_ is the forward reaction rate (1 molecule^−1^ s^−1^), *C*_*b*_ represents the density of bound ligands, *C*_*L*_ the original density of the ligands (molecules area^−1^), and *A*_*L*_ is the local area of a single fiber associated with the integrin node under consideration. Note that (*C*_*L*_-*C*_*b*_) represents the number of unbound ligands available for bonding in the vicinity of the integrin node. In simulations, values of *A*_*L*_ *C*_*L*_ were identically set to be 300 molecules per a ECM fiber node in three ECM fibers network models. Once in the tugging phase, the strength of the FCs is tested (rupture test), and the phase switches to the contractile phase if the bond does not rupture and the tension rises beyond a specified threshold level at the filopodial tip (3–4 nN/μm) [36]. If it ruptures, it switches back to the outgrowing phase. The formation of FCs is restricted to the proximal tip of the filopodia, since it is known that veil advance typically results from the formation of FCs proximal to the tip of the filopodial shaft [32] (Fig 1A and 1E, S3 Video).

### Geometrical model of contractile filopodia

The filopodial model is geometrically composed of *N*_*AM*_ compartments of acto-myosin (AM) assemblies; the first compartment is attached to the root of filopodium and the last compartment is connected to the tip of filopodium (Fig 3A and 3B). We model filopodial contractile motion in a manner characterized by that of actin stress fibers (SF) [27]. The stiffness of an AM assembly is variable, $\kappa_{AM,j}=\frac{E_{AM}A_{AM}}{L_{AM,j}^{1}},\;j=1..N_{AM}$ where *E*_*AM*_ is the Young’s modulus of AMs [37], *A*_*AM*_ is the average cross-sectional area of AMs in a filopodium and $L_{AM,j}^{1}$ is an unstressed length of a single compartment of the *j*-th AM. The length of each compartment contracts at both ends according to the myosin II sliding rate, *v_m,j_*. Therefore, $\frac{dL_{AM,j}^{1}}{dt}=-2v_{m,j}$. Furthermore, it is known that myosin motors slides on actin filaments in opposite directions of FC forces at the filopodial tip [4]. That is, an increasing elastic load from the ECM is transmitted through FCs at the tip of the filopodium, through the AM assembly, to the myosin motors, which, in turn, decreases the myosin speed (*v*_*m*_). It has been known that myosin sliding velocities affected by ECM stiffness, that is, myosin sliding speed is high on the soft gel but it is low on the hard gel [4]. In addition, measurements on few or many myosin molecules by optical trap [38] reported that the force-velocity relationship for these molecules is significantly similar to that of whole muscle [39] or muscle fibers [40,41]. To incorporate these characteristics into the filopodia dynamics, we adopt the force-velocity relationship for the whole muscle [39] into the following equation: $v_{m}=v_{m0}\frac{F_{stall}-F_{TR}}{F_{stall}+c_{m}F_{TR}}$ where *v*_*m*0_ is the sliding rate of myosin in the absence of load (10 nm/s) [42], *F*_*stall*_ is the stall force of 1 nN, *c*_*m*_ is a dimensionless myosin parameter of 0.1, and *F*_*TR*_ is the sensed elastic force from the ECM at the tip of filopodium. It should be noted that the speed of myosin become zero when *F*_*TR*_ exceeds *F*_*stall*_, and linear relationship of force-velocity can be tuned to that of the nonlinear relationship by increasing the value of *c*_*m*_ more than 1.

The total elastic energy stored in the AMs in the filopodium is given by

$${\text{H}}_{AM}^{}=\sum_{j=1}^{N_{AM}}\left[\frac{\kappa_{AM,j}^{}}{2}{\left(d_{AM,j}-L_{AM,j}^{1}\right)}^{2}\right]$$ (5)

where *d* *_AM,j_* represents the distance of the *j*-th contractile AM compartment under tension (Fig 3B). Using the virtual work theory, forces due to contractile myosin motor activity at the *j*-th node of filopodial shaft is given by

$$F_{AM,j}^{f}=-\frac{\partial {\text{H}}_{AM}^{}}{\partial x_{j}^{f}}=-\kappa_{AM,j}^{}\left(d_{AM,j}-L_{AM,j}^{1}\right)\frac{\partial d_{AM,j}}{\partial x_{j}^{f}}+\kappa_{AM,j+1}^{}\left(d_{AM,j+1}-L_{AM,j+1}^{1}\right)\frac{\partial d_{AM,j+1}}{\partial x_{j+1}^{f}}.$$ (6)

#### Characterization of three ECM fiber network models

It is known that mechanical properties of collagen gels depend on the pH of the collagen solution during polymerization [43]: gels become stiffer and structures of gels were found to contain both decreased fiber diameters and pore sizes as the polymerization pH is increased. Accordingly, we built three different models of ECM fiber networks comprising of three different pore sizes of 0.5, 1.0 and 1.5 μm and three different fiber diameters of 28, 34 and 41 nm, respectively (S3 Fig and S4 Video). First, we aimed to simulate mechanical stretching tests for the three ECM fiber network models in order to characterize and compare the three models with experimentally measured bulk moduli of ECM gels [43]. Various simulations of mechanical stretching tests for each ECM fiber network model were performed using two parameters of single fiber diameters and moduli (S4–S6 Figs and S1 Text). We found that the moduli of the model are linearly and significantly increased as single fiber diameter or single fiber modulus is increased in each ECM model (S3A and S3B Fig). Both simulation and experiment show an excellent agreement over both ECM fiber diameters of 28, 34 and 41 nm and ECM pore sizes of 0.5, 1.0 and 1.5 μm. Statistical analysis of linear regression was performed by comparing the experiment and the simulation in terms of the mean values of modulus for the same conditions of fiber diameter and ECM pore size. Good correlations were found between the two with R^2^ = 0.898 (S3C Fig). It should be noted that identical fiber modulus of 1 MPa for all the ECM models gives the best agreement with the experiments. Moduli of ECM gels are strongly dependent on single fiber diameter and ECM pore size, and the above fiber network model successfully verifies these properties.

In literatures, measured single fiber moduli and diameters in aqueous media have been reported to be from 32 to 800 MPa, and from 40 to 1000 nm, respectively [44–46]. Interestingly, Graham et al. showed a peak value of 32 MPa at the larger strains than 4 and the lowest strains ~2 MPa at the range of strains between 0 and 2 using a fiber diameter of 40 nm [44], and our simulated fiber diameters and modulus are in a good agreement with their experimental data at the low strains <1 since we assume the cell can generate traction force interacting with fibers at this low strain regime. In addition, an image-based multiscale modeling technique used fitting parameters of fiber modulus of 6.7 MPa and a fiber diameter of 100 nm to predict both tissue-level and network-level fiber reorganization [47].

#### Prediction of 3D cell-ECM interactions and penetration speeds and comparison with experimental observations

**Fig 4:**
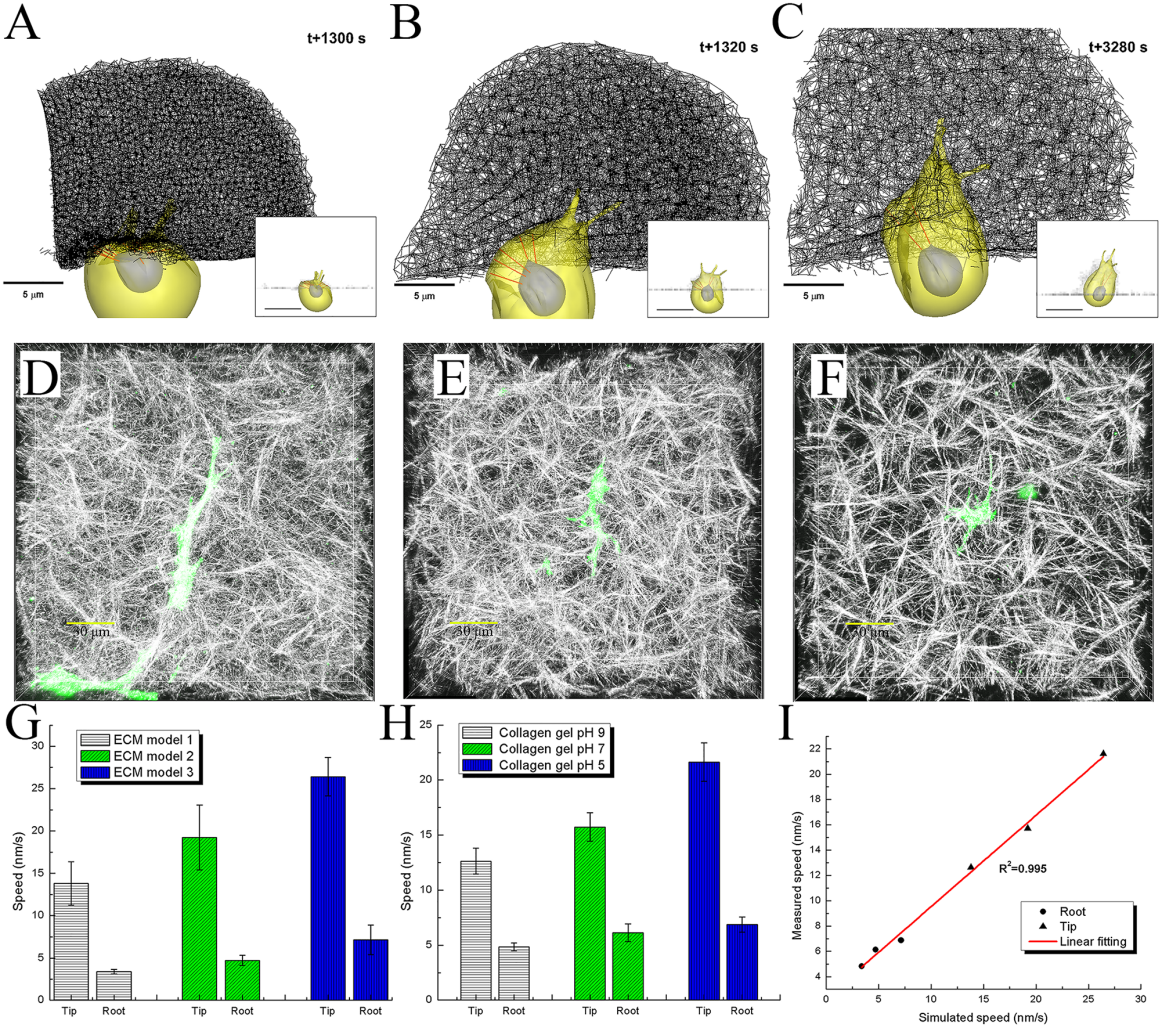
Comparison of filopodia penetration dynamics between simulations and experiments. Simulated cell invasions into three different ECM fiber network models with pore sizes of **A**) 0.5, **B**) 1.0, and **C**) 1.5 μm. 3D confocal images of GFP transfected single cell migration in three different collagen gels with pH levels of **D**) 9, **E**) 7, and **F**) 5. Yellow scale bars indicate 30 μm. Note that three ECM fiber network models with pore sizes of **A**) 0.5, **B**) 1.0, and **C**) 1.5 μm correspond to three different collagen gels with pH levels of **D**) 9, **E**) 7, and **F**) 5, respectively. **G**) Bar graphs showing simulated average speeds and standard error of means (N = 5) at both tip and root of filopodium for the three different ECM fiber network models. Note that three different single fiber diameters of 41, 34 and 28 nm are used for three ECM models with pore sizes of 1.5, 1.0 and 0.5 μm, respectively. **H**) Bar graphs showing experimentally measured average speeds and standard error of means (N = 18, 11, and 17 for three collagen gels for two sets of experiments) at both tip and root of filopodium for three collagen gels. Error bars in **G**) and **H**) indicate standard error of mean. **I**) Liner regression (R^2^ = 0.995) between simulated speeds of both tip and root of filopodium in **G**) and experimentally measured speeds of both tip and root of filopodium in **H**).

Following successful characterizations of the mechanical properties of the three ECM models with collagen gel experiments [43], simulations of filopodia penetration and cell invasion were performed using these three ECM models with three different pore sizes of 0.5, 1.0 and 1.5 μm and three different fiber diameters of 28, 34 and 41 nm (Fig 4A, 4B and 4C, S5–S7 Videos) to predict coordinated filopodia behaviours and cell-ECM interactions in 3D. This allowed us to investigate the dynamic interplay between filopodial traction generation and ECM gel remodelling in 3D in a quantitative manner.

Furthermore, for comparison and validation of the model, experiments were performed using HUVECs transfected with cytosolic GFP migrating in the three types of collagen gels with pH levels of 9, 7, and 5 (Fig 4D, 4E and 4F, S8–S10 Videos) filled in-vitro microfluidic device [48]. Penetration speeds were measured at both tip and root of each filopodium located on the leading edge of the cell. Each experiment was recorded at a time-interval of five minutes over 30–60 minutes, while simulations were conducted with a time-interval of one second over 10–20 minutes for the three gel types (Fig 4A, 4B and 4C). For an example, the time-averaged speed at the filopodial tip,$v_{mean}^{tip}$, is expressed as following:

$$v_{mean}^{tip}=\frac{1}{t_{f}-t_{0}}\int_{t_{0}}^{t_{f}}v_{}^{tip}\left(t\right)dt=\frac{1}{t_{f}-t_{0}}\int_{t_{0}}^{t_{f}}ds_{}^{tip}\left(t\right)\approx \frac{1}{t_{f}-t_{0}}\sum_{i=1}^{N_{sample}-1}{\left({\left(x_{i}^{tip}-x_{i-1}^{tip}\right)}^{2}+{\left(y_{i}^{tip}-y_{i-1}^{tip}\right)}^{2}+{\left(z_{i}^{tip}-z_{i-1}^{tip}\right)}^{2}\right)}^{1/2}$$ (7)

where *t*_0_ is the initial time, *t*_*f*_ is the final time, and *N*_*sample*_ is the number of samples.$x_{i}^{tip}$,$y_{i}^{tip}$, and $z_{i}^{tip}$ are coordinates of filopodium at the *i*-th time-interval.

We have found that speeds of both tip and root of filopodia increase as the pore size is increased in experiments (Fig 4H). The simulated speeds of both tip and root of filopodia, too, shows a trend similar to the experiments (Fig 4G). The simulations and experiments have shown an excellent agreement for all the ECM pore sizes of 0.5, 1.0 and 1.5 μm (or pH levels of 9, 7, and 5). Statistical analysis of linear regression was performed for comparing the experiments and simulations in terms of the mean filopodial tip speed as well as of the mean root speed under the same conditions of ECM pore size (or pH level). High correlations were found between the two, with R^2^ = 0.995 and a slope of 0.7 (Fig 4I).

#### Filopodia state dynamically alters during penetration

**Fig 5:**
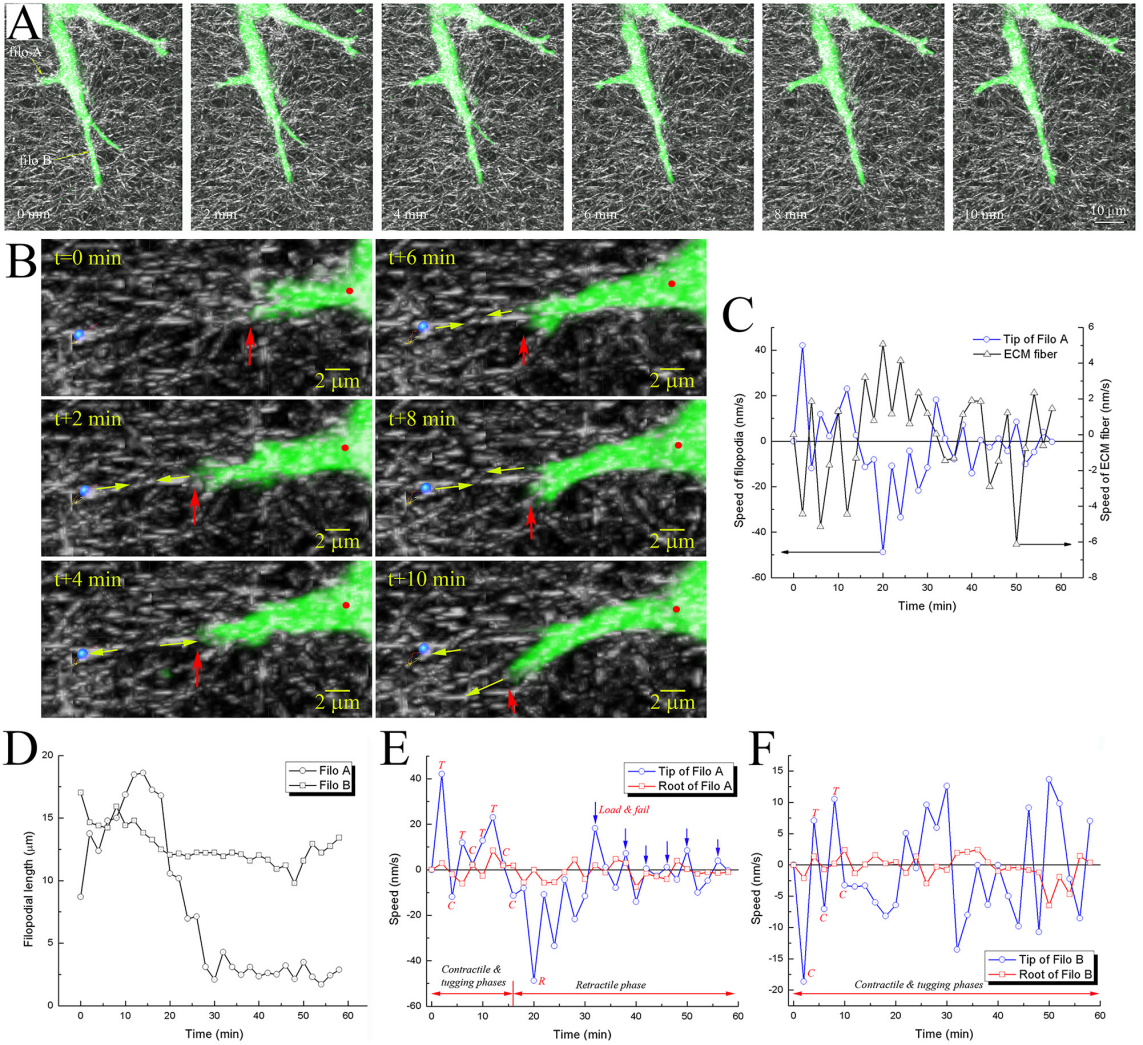
Experimental observations of filopodia state changes during penetration. **A**) 3-D confocal images showing filopodia protrusive, tugging, and contractile motions in GFP-transfected HUVECs, and remodeling of collagen fiber network at time points of 0, 2, 4, 6, 8 and 10 minutes. **B**) 3D collapsed images showing the crawling behavior of filopodial tip at time points 0, 2, 4, 6, 8 and 10 minutes. Blue sphere indicates a monitored location on the ECM, which was shown to be mechanically linked to the filopodial tip. Yellow arrows indicate directions of displacements of the blue sphere and the filopodial tip. Red arrows and red dots indicate filopodial tips and roots, respectively. **C**) Graph showing temporal variations of speeds at the tip of filopodium (Filo A in **A**) and blue sphere in **B**). **D**) Graphs showing filopodial length changes in Filo A and B over time. Graphs in **E)** and **F)** showing temporal variations in speedsn at both tip and root of the two filopodia: Filo A in **E**) and Filo B **F**). Note that plus and minus signs represent forward and backward movements of filopodium, respectively, and blue arrows in **E**) indicate fast oscillatory ‘load-and-fail’ traction dynamics during the retractile phase. *T*, *C*, and *R* in **E**) and **F**) indicate tugging, contractile, and retractile phases, respectively.

Experimental observation revealed additional behaviours of filopodia that support the mechanisms identified in our filopodia dynamic model as well as those of prior works [4–10]. Filopodial protrusive and contractile motions were recorded simultaneously with ECM fibers deformation and remodelling (Fig 5A and S11 Video). An example can be seen in Fig 5A: two filopodia extending in different directions and their proximal ECM fibers were tracked over time: ‘Filos A’ and ‘Filos B’. Filo A started crawling on collagen fibers at time 0, its tip was further protruded and branched along multiple collagen fibers in different directions (2 min, tugging phase); collagen fibers were stretched due to the contractile motion of filopodia (4 min, contractile phase); following that, fluctuating motions at the filopodial tip was further observed (6 min– 10 min, tugging phase).

Movements of the filopodium and those of a few specific ECM fibers near the filopodium were highly correlated, indicating the existence of linkage between the two. To investigate this correlation in movements, the background fiber images were eliminated from the original 3D confocal images and displacements of both filopodium tip and neighbouring fibers were tracked over time (Fig 5B and S12 Video). Fig 5C shows how the speed of this ECM fiber marked with a blue sphere is completely synchronized with the speed of the filopodial tip for the first 30 min. Note that plus and minus signs represent forward and backward movements of filopodium. In fact the filopodial tip crawled along this particular fiber; tugging the fiber bit by bit (t+2, t+6, and t+8 min) induced the fiber motion that was completely out of phase with the filopodium (Fig 5C). Interestingly, at t+10 min, we can notice relaxation of the fiber (blue sphere displaces away from the filopodium tip), which is correlated with the filopodium tip switching from that fiber to an adjacent fiber (t+10 min, bottom yellow arrow).

Filopodia state changes were also observed in these experimental data. The filopodium was rapidly retracted at t+18 min (S12 Video and Fig 5D), and fast oscillatory ‘load-and-fail’ traction dynamics were observed at t+32 min (blue arrows in Fig 5E). Plotting the tip speed together with the speed of the filopodium root, which is approximately the same as the speed of veil (cell membrane advance) (Fig 5E); we can find how filopodia dynamics influences the cell advance. In addition, Filo B also showed similar behaviours except for the rapid retraction of Filo A (Fig 5F). The migratory trajectory of the cell indicated that Filo B was at the leading edge of the cell and had been polarised in that direction. As a result, its life span appears to be longer, exhibiting slower contractile motion rather than rapid retraction as in the case of Filo A (Fig 5E and 5F).

#### Comparisons of traction fields in ECM fiber network

**Fig 6:**
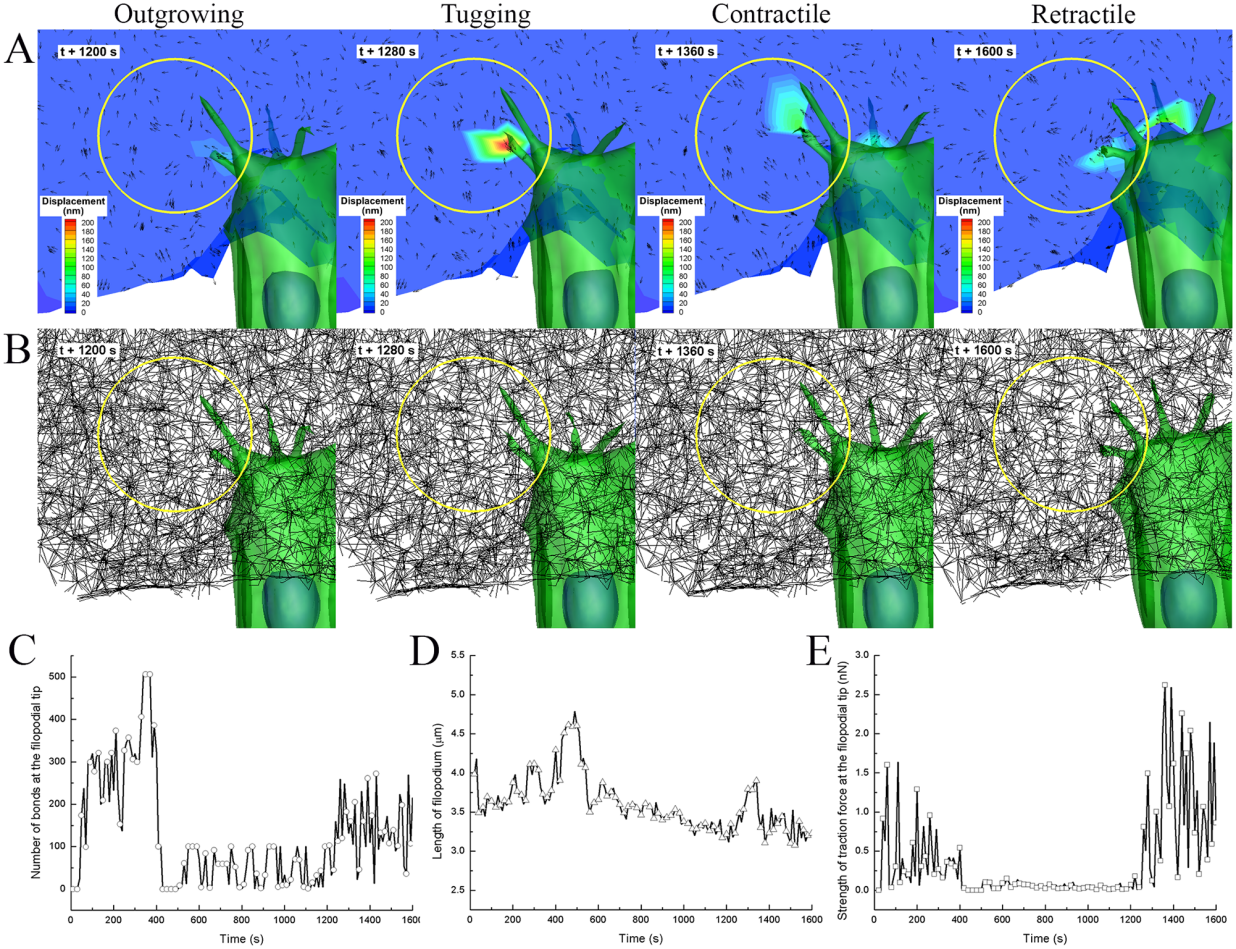
Traction simulation of ECM fiber network model with pore size of 1.5 μm. Selected examples of **A)** sectional contours and vector plots of ECM fiber displacement, and **B)** clipped view of ECM fibers network at different time points of 1200, 1280, 1360, and 1600 seconds, respectively. Temporal variations of **C)** number of receptor-ligand bonds at the filopodial tip, **D**) filopodial length, and **E**) the magnitude of traction force at the filopodial tip which is mechanically interact with ECM fibers.

Distinct behaviours of filopodial motion have been confirmed using a 3D cell migration microfluidic assay in which 200 nm-sized fluorescent beads are embedded into the collagen gel. As a result, beads move towards the extending filopodium tip during the tugging and contractile phases, and away from the filopodium during retractile motion (S13 and S14 Videos). Based on these experimental observations, computational model of filopodia penetration dynamics is validated by showing similar motions of ECM fibers toward or away from the filopodium during the tugging and contractile phases or retractile phase, respectively. These imply that ECM fibers repeatedly underwent tension and relaxation; the direction of displacement vectors changed accordingly, switching between pointing towards the filopodia and pointing away from it cyclically (Fig 6A and S12 Fig and S15 Video). Furthermore, the number of receptor-ligand bonds at the tip of filopodium, the length of filopodium, and the strength of traction force at the tip of filopodium were computationally measured, as shown in Fig 6C, 6D and 6E.

At the time point of 1200 s (outgrowing phase), the filopodium was found to start to protrude as its length was increased from 3.2 μm, the magnitude of elastic force ECM fibers, where filopodial tip are adhered, was found to be lowest peak as 72 pN (Fig 6A and 6B). It should be noted that simulated data were sampled at every ten seconds. In addition, lowest displacement fields of ECM fibers were observed at the local ECM area as the filopodia generate weak traction force through its outgrowing motion. Interestingly, directions of displacement fields are found to be away from the filopodia, which means ECM fiber network are relaxed.

At the time point of 1280 s (tugging phase), the filopodia was appeared to tug neighboring ECM fibers as its length was growing from 3.37 μm to 3.53 μm, the magnitude of elastic force ECM fibers, where filopodial tip are adhered, was found to be a high peak as 1.49 nN and the number of receptor-ligand bonds at filopodial tip was also found to be high as 248. In addition, highest displacement fields and substantial deformations of ECM fibers were observed at the local ECM area as the filopodia generate traction force through its tugging motion (Fig 6A and 6B and S12 Fig). Interestingly, directions of displacement fields are found to be towards the filopodia, which means ECM fiber network are tensioned.

At the time point of 1360 s (contractile phase), the filopodia was appeared to rapidly contract from 3.9 μm to 3.3 μm, the magnitude of elastic force ECM fibers, where filopodial tip are adhered, was found to be highest peak as 2.62 nN. In addition, highest displacement fields and substantial deformations of ECM fibers were observed at the local ECM area as the filopodia generate traction force through its contractile motion (Fig 6A and 6B). Interestingly, directions of displacement fields are also found to be towards the filopodia, which means ECM fiber network are tensioned.

At the time point of 1600 s (retractile phase), the filopodium was found to retract as its length was decreased to 3.2 μm, but the magnitude of elastic force ECM fibers was appeared to be a low value of 0.86 nN, and the number of receptor-ligand bonds was also moderately decreased to 215. Distinct behaviour of filopodial retractile motion is found to be the relaxation of tensioned ECM fiber network as the filopodia switches its motion from the contractile phase to retractile phase, which resulted in directions of displacement fields are observed to be away from the filopodium (Fig 6A and 6B and S12 Fig).

## Discussion

It has been reported that distinct load-fail behaviour and frictional slippage behaviour of filopodia at both soft and hard gels can be explained by using the motor-clutch mechanism at the filopodial shaft [4]. Furthermore, the speed of the filopodia retraction is highly dependent on the stiffness of ECM surrounding the filopodia tip: as the stiffness of the ECM increases, the retrograded flow of actin at the filopodial shaft becomes faster, but the traction stress generated in the ECM becomes lower [4]. In the current stochastic model of filopodia penetration dynamics, these filopodia behaviours have been reproduced and their mechanisms have been elucidated as a dynamic 3D interplay between filopodia traction and ECM remodelling (S5–S7 Videos). Filopodia mechanically interact with ECM fibers to cause gel compaction and fiber remodelling [24] (S19 Video). In turn, the local stiffness of the ECM, which is modulated by the filopodia activities, influences the contractile/retractile behaviours of the filopodia via actin motor activities. Here, the contractile motion differs from the retractile motion in that the contractile motion leads to the generation of traction fields in the ECM surrounding the filopodia via the motor-clutch mechanism, while the retractile motion results in the relaxation of filopodia as well as the relaxation of ECM fibers. Furthermore, these distinct behaviours of filopodial motion have been confirmed using a 3D cell migration microfluidic assay in which 200 nm-sized fluorescent beads are embedded into the collagen gel. As a result, beads move towards the extending filopodium tip during the tugging and contractile phases, and away from the filopodium during retractile motion (S13 and S14 Videos).

The ECM stiffness is known to increase as the concentration of crosslinking molecules increases [49]. On the other hand, an increase in the number of crosslinks leads to a decrease in the ECM pore size. In our current study, three kinds of ECM fiber network models were built with pore sizes of 0.5, 1.0 and 1.5 μm and single fiber diameters of 28, 34 and 41 nm, respectively, based on mechanical properties experiments [43]. The line stiffness of a single fiber $\left(=E_{f}^{e}A_{f}^{}/L_{f}^{e0}\right)$ with a pore size for the three different ECM models were calculated to be 0.880, 0.908 and 1.2 pN/nm, respectively. Although these values are within the range of soft substrate whose stiffness is less than 1 pN/nm, apparent values of stiffness for the fiber network at the local area of 1 μm^2^ are significantly increased to the range of harder substrate whose stiffness is more than 10 pN/nm as pore size becomes smaller and more crosslinks are added. To promote cell migration in a 3D ECM fiber network with the smallest pore size, degradation of the ECM fiber network is required [50]. For comparison we simulated filopodial penetration models with no degradation of the ECM fiber network, that is, MT1-MMP deficient cell model (S16 Video). This resulted in inhibition of the cell invasion into the ECM fiber network, compared to that observed for deep cell invasion model that incorporates filopodia penetration dynamics and ECM fiber network degradation (S7–S10 Figs and S2 Text and S17 Video). Thus, our simulated results reveal that the degradation of ECM fiber network plays an important role in filopodia penetration dynamics during both tugging and contractile phases. Without ECM degradation, filopodia can still grab ECM fibers; however, the lamella, where the root of the filopodial shaft is connected to the cellular membrane, can hardly penetrate through the ECM fiber network. With the addition of the component of degradation by MMP-2, our ECM fiber network model is able to simulate more complex situations; local fiber network, where MMP-2 is diffused from the cellular membrane, gradually becomes less stiffer since degraded fiber network is decomposed into multiple elements of single fibers whose stiffness are less than 1 pN/nm. Thereby, the lamella can easily penetrate through the degraded ECM fiber network.

Filopodia dynamics is very complex, and many other factors are likely to contribute to these dynamics. We have applied the Bell model to our filopodial focal complex model to rupture bonds between integrins and collagen molecules with force, and it is known as the ‘slip bonds’ as the ligand should slip out of the binding pocket more rapidly under higher tensile force [35]. As results, probability of rupturing an individual bond becomes higher and the lifetime of its bond becomes shorter under higher tensile force ~200 pN. On the other hand, some cell-ECM interactions have been recently known to indicate ‘catch bonds’ behavior [51,52]; the lifetime of catch bonds show biphasic distributions under applied force, which implies that the lifetime of catch bond takes a maximum at a critical value of applied force. It is of interest how catch bonds likely to influence filopodia dynamics. There was an interesting work of stochastic models comparing behaviors between ‘catch bond’ and ‘slip bond’ in relation to actin retrograde flow and corresponding traction stresses [53]. In their work, in case of ‘slip bond’, bond fraction was rapidly decreased as actin retrograde speed was increased. However, in case of ‘catch bond’, bond fraction takes a maximum at actin retrograde speed of 5 nm/s. Overall, ‘catch bond’ case showed higher bond fraction than ‘slip bond’ case at the range of actin retrograde speed > 5 nm/s. Based on these results, filopodia dynamics with ‘catch bonds’ is likely influence longer lifetime focal complex formations than that with ‘slip bonds’. Furthermore, filopodia dynamics with ‘catch bonds’ is likely influence stronger traction fields in ECM than that with ‘slip bonds’.

The initial goal of current model was aimed at understanding how filopodia penetration dynamics plays an important role in 3D cell invasion into an ECM fiber network. The ultimate goal of the future cell model will be the development of the model like a realistic cell by capturing complicated morphology changes the cell. However, comparisons of cellular morphologies between the simulated cell models and experimentally measured HUVECs were limited in current cell model because morphologies of simulated cells were rounded or oval shapes, but those of experimentally measured cells were very elongated. To our knowledge, this discrepancy was resulted from two assumptions of the model; 1) the number of nodes in the cellular membrane was set to be 549, and 2) maximum number of clustered integrins per a node was set to be 100. Therefore, the direction of the future cell migration model will be to improve the morphology of cell model by increasing the number of nodes > 10,000 as the size of a mesh size is close to molecular size of ~80 nm, and decreasing the number of clustered integrins. In addition, all diffusion coefficients and some secretion rates of biochemical concentrations in the current model were assumed to be identical for three ECM fiber models. However, in fact, heterogeneous diffusion coefficients and secretion rates of biochemical concentrations should be considered in the future cell migration model since recent experimental observations have indicated that diffusion coefficient of bovine serum albumin (BSA) is decreased as the ECM is stiffer and a pore sizes of ECM network is reduced more [22]. Furthermore, the future cell model can be extended to more complex models of cancer metastasis including a collective migration mediated by both cell-cell and cell-ECM adhesions, epithelial-mesenchymal transition (EMT)-mediated mesenchymal cell migration, amoeboid migration in ECM [54].

## Model

### Intracellular mechanics

The structural mechanics and intracellular mechanics are other key mechanisms involved in cell invading into 3D gel. Here, we formulate them by extending the previous dynamic model for cell migration on curved surfaces [26 **]**, and cell migration and spreading on planar micropatterns [27 **]**. The essential equations in the model include: 1) an equation for FA dynamics based on Monte-Carlo simulations of ligand-receptor bonds, 2) two equations for deformations of double elastic membranes: an outer cell membrane and an inner nuclear membrane, 3) an equation describing the contractile motion of actin stress fibers, which is extended from FAs on the cortical surface to the nuclear membrane, and 4) lamellipodium protrusion by actin polymerization [19] with a constant force of 300 pN. The detailed description of the equations in the model can be found in previous works [26,27 **]**. Among them, the major extension in the mechanobiological dynamics model presented here is FAs dynamics in 3-D ECM fiber network model (Fig 1B). The FA force acts between the *i*-th integrin node on the cellular membrane and points of ECM fibers where the extension of the unit vector normal to the cellular membrane interacts with the nearest point of ECM fibers.

### Construction of ECM fiber network model

To generate the computational model for ECM fiber network, a 3D cubic structure (50 × 50 × 50 μm) was initially drawn using AUTOCAD software. This structure was made in a stereolithography (STL) file format. Then, the surface geometry of the STL file was imported into a commercial CFD software package (STAR CCM++, CD-adapco) to build tetrahedral grids for the model. As a preprocess, further refinements to the STL file, including surface triangulation, tetrahedral volume meshing, and optimization for computational stability, were carried out to build three different ECM fiber network models with pore sizes of 0.5, 1, and 1.5 μm (S11A Fig). These preprocessed tetrahedral grids for three different ECM fiber network models with pore sizes of 0.5, 1, and 1.5 μm consisted of ~ 3.0×10^6^, 3.8×10^5^, and 1.3×10^5^ elements, respectively. Finally, these grids were modified to construct components of ECM fiber network, such as elastic fibers and crosslinks (S11B Fig). Here, red and yellow nodes in these grids represent crosslink and fiber nodes, respectively. To construct the fiber network, each line between two red nodes was divided by *N*_*div*_ fiber segments and two crosslink segments that consist of *N*_*div*_ +1 fiber nodes (yellow). Here, values of *N*_*div*_ for ECM fiber network models with pore sizes of 0.5, 1, and 1.5 μm were respectively set to be 1, 2, and 3 with an assumption that the density of collagen molecules along all fibers in three ECM fiber network models was identical (300 collagen molecules per a fiber node), and the distance of a crosslink segment was set to be 30 nm. After segmentations of fibers, one set of fiber segments were randomly made to connect to coaxial neighbouring sets of fiber segments under a condition when the angle between two connected sets of fiber segments was above 60° (S11C Fig). In addition, the number of connected fibers at the *i*-th crosslink node was set to be $P_{f}\left(\frac{N_{i}^{fs}}{2}\right)$, where *P*_*f*_ is an initial ratio of forming fibers at the *i*-th crosslinks node (0.7), and $N_{i}^{fs}$ is a total number of linked fiber segments at the *i*-th crosslinks node.

### ECM fiber network dynamics

**Fig 7:**
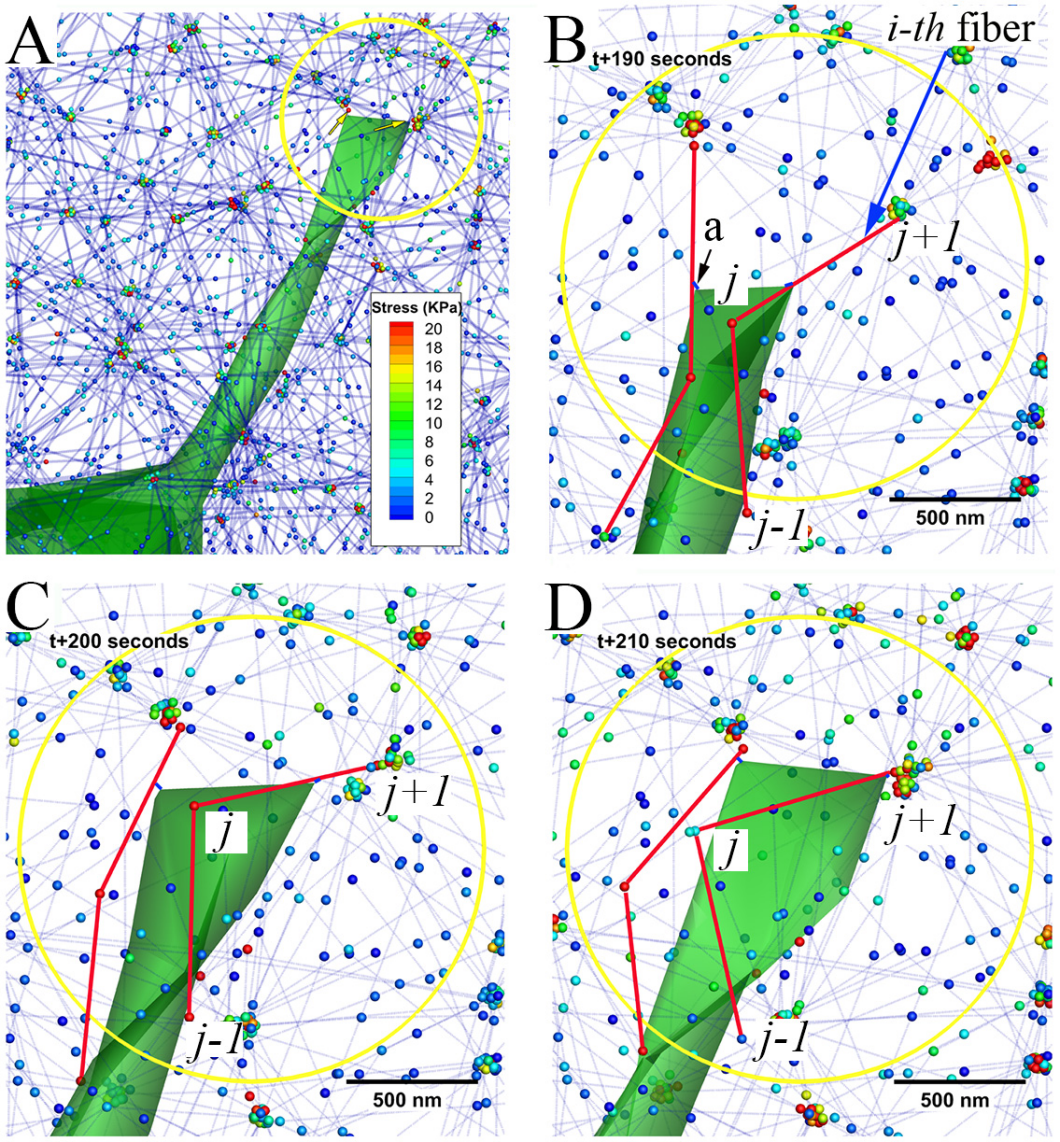
Filopodial tugging phase during the simulation. **A**) One instant during the simulation of a filopodium interacting with an ECM fiber network model; blue dotted lines represent fibers and spheres represents two kinds of ECM nodes (fiber node and crosslink node); colours of spheres indicate magnitudes of stress at ECM nodes; yellow arrows indicate directions of FC movements. **B)**, **C)**, and **D)** indicate timeframe shots of filopodial tugging phase showing movements of two FCs (tiny blue bars; ‘a’ in **B**) at the *k*-th filopodial tip along two different fibers (thick red lines) and substantial pulling ECM fibers towards the filopodial tip at t+190, t+200 and t+210 seconds, respectively. Note, **B)**, **C)**, and **D)** are magnified views in the circle marked in **A)**, and scale bars indicate 500 nm.

We assume the ECM fiber network to be composed of elastic ECM fibers and crosslinks, which make strong bonds between adjacent fibers [13]. The elastic energy stored in the ECM fiber network can be expressed in terms of the stretching and bending properties of the constituent fibers. The stretching modulus of a fiber is given by $\kappa_{f,s}^{e}\left(=E_{f}^{e}A_{f}^{}\right)$, where $E_{f}^{e}$ and $A_{f}\left(=\pi r_{f}^{2}\right)$ are the Young’s modulus (1 MPa) and the cross-sectional area of a single fiber, respectively. The bending modulus of a fiber is given by $\kappa_{f,b}^{e}\left(=E_{f}^{}I_{f}\right)$, where $I_{f}\left(=\pi r_{f}^{4}/4\right)$[55]. The stretching elastic energy of the *j*-th segment of the *i*-th fiber is given as a function of the difference between the stressed ($L_{i\;j}^{e}$) and unstressed ($L_{i\;j}^{e0}$) lengths, and the bending elastic energy as the one of stressed ($\theta_{i\;j}^{e}$) and unstressed ($\theta_{i\;j}^{e0}$) angles at the *j*-th node between two segments in the *i*-th fiber (Fig 7). The total elastic energy in the *i*-th ECM fiber in the network can be expressed as following:

$${\text{H}}_{f,i}^{e}=\frac{\kappa_{f,s}^{e}}{2}\sum_{j=1}^{N_{i}^{e}}\frac{{\left(L_{i\;j}^{e}-L_{i\;j}^{e0}\right)}^{2}}{L_{i\;j}^{e0}}+\frac{\kappa_{f,b}^{e}}{2}\sum_{j=1}^{N_{i}^{e}}\frac{{\left(\theta_{i\;j}^{e}-\theta_{i\;j}^{e0}\right)}^{2}}{L_{i\;j}^{e0}}.$$ (8)

Here, it should be noted that the elastic energy at the *j*-th node in the *i*-th fiber is summed only for coaxial neighbouring nodes. Similarly, the elastic force at the *j*-th node in the *i*-th fiber,$F_{E,i\;j}^{e}$, can be derived by using the virtual work theory:

$$F_{E,i\;j}^{e}=-\frac{\partial {\text{H}}_{f,i}^{e}}{\partial x_{i\;j}^{e}}=-\kappa_{f,s}^{e}\sum_{k=j}^{j+1}\frac{\left(L_{i\;k}^{e}-L_{i\;k}^{e0}\right)}{L_{i\;k}^{e0}}\frac{\partial L_{i\;k}^{e}}{\partial x_{i\;j}^{e}}-\kappa_{f,b}^{e}\sum_{k=j-1}^{j+1}\frac{\left(\theta_{i\;k}^{e}-\theta_{i\;k}^{e0}\right)}{L_{i\;k}^{e0}}\frac{\partial \theta_{i\;k}^{e}}{\partial x_{i\;j}^{e}}$$ (9)

where $\theta_{ik}^{e}={\text{cos}}^{-1}\left({\hat{t}}_{i,k}\cdot {\hat{t}}_{i,k+1}\right),$ ${\hat{t}}_{i,k}$ and ${\hat{t}}_{i,k+1}$ are tangential unit vectors at the *k* and *k*+1-st segments in the *i*-th fiber, respectively, and $\frac{\partial \theta_{f,i\;k}^{e}}{\partial x_{i\;j}^{e}}=\frac{-1}{\sqrt{1-{\left({\hat{t}}_{i,k}\cdot {\hat{t}}_{i,k+1}\right)}^{2}}}\left(\frac{\partial {\hat{t}}_{i,k}}{\partial x_{i\;j}^{e}}\cdot {\hat{t}}_{i,k+1}+{\hat{t}}_{i,k}\cdot \frac{\partial {\hat{t}}_{i,k+1}}{\partial x_{i\;j}^{e}}\right).$

To incorporate the degradability factor into the ECM fiber network and its nonlinear behaviour under mechanical responses, we consider that each crosslink node comprises crosslink molecules, such as amino acids, that can rupture. Fig 7B shows an example of connectivity between the *i*-th crosslink and the two neighbouring fibers. We model the degradability of ECM fiber network by considering detachment events among the *i*-th crosslink node and its neighbouring fibers, and the degradability of ECM fiber network depends on a local value of the ECM integrity ($I_{ECM,i}=C_{ECM,i}/C_{ECM,i}^{0};\;0\leq I_{ECM,i}\leq 1$) (see S18 Video). Here, *C* *_ECM,i_* and $C_{ECM,i}^{0}$ are concentrations of the *i*-th ECM node at present and initial states (10μM), respectively. The number of uncrosslinked (or degraded) fibers at the *i*-th crosslink node,$N_{i}^{uf}$, is calculated as $N_{i}^{uf}=\left(1-I_{ECM,k}\right)N_{i0}^{f}$, where $N_{i0}^{f}$ is an initial number of crosslinked ECM fibers at the *i*-th crosslinks node.

### Reaction-diffusion mass transfer in 3D ECM

To incorporate chemical interactions of 3D ECM with filopodia and cellular membranes (Fig 1C), four distinct dynamics associated with chemotaxis, proteolysis, haptotaxis, and degradation are modelled. Seven reaction-diffusion equations for concentrations of VEGF, MMP-2, TIMP-2 (S7 and S8 and S9 Figs), MT1-MMP, a ternary complex of MT1-MMP:TIMP-2:proMMP-2 [14], ligands (or collagen molecules) and ECM are numerically solved using Finite Volume Method (FVM) [56]. Constitutive equations for the seven biochemical concentrations are summarised in Table 3. In particular, MT1-MMP and TIMP-2 secretions at the membrane near the roots of filopodia are modelled as source terms [25].

**Table 3:** Details of RD module.

*X*_1*i*_	*R*_*X*1_*i*__ from the equation: $\frac{\partial C_{X_{1i}}}{\partial t}=\nabla \cdot \left(D_{X_{1i}}\nabla C_{X_{1i}}\right)+R_{X_{1i}}$
VEGF	$-k_{VEGF}^{decay}C_{VEGF}$
MMP-2	$-k_{TIMP2:MMP2}^{on}C_{TIMP2}C_{MMP2}+k_{Complex:MT1-MMP}^{on}C_{Complex}C_{MT1-MMP}-k_{MMP2}^{decay}C_{MMP}$
TIMP-2	$-k_{TIMP2:MMP2}^{on}C_{TIMP2}C_{MMP2}\;-k_{TIMP2:MT1-MMP}^{on}C_{TIMP2}C_{MT1-MMP}+k_{Complex}^{off}C_{Complex}+\alpha_{TIMP2}\left(x_{base}^{f}\right)C_{Ligand}$
Ligand	$-k_{Ligand}^{decay}C_{Ligand}+k_{ECM}^{deg}C_{MMP2}C_{ECM}$
*X*_2*i*_	$R_{X2_{i}}$ from the equation: $\frac{\partial C_{X_{2i}}}{\partial t}=R_{X_{2i}}$
MT1-MMP	$-k_{TIMP2:MT1-MMP}^{on}C_{TIMP2}C_{MT1-MMP}+k_{Complex}^{off}C_{Complex}-k_{MT1-MMP}^{decay}C_{MT1-MMP}+\alpha_{MT1-MMP}\left(x_{base}^{f}\right)C_{Ligand}$
Complex	$k_{TIMP2:MT1-MMP}^{on}C_{TIMP2}C_{MT1-MMP}+k_{Complex:MT1-MMP}^{on}C_{Complex}C_{MT1-MMP}-k_{Complex}^{off}C_{Complex}$
ECM	$-k_{ECM}^{deg}C_{MMP2}C_{ECM}$

$k_{VEGF}^{decay}$,$k_{MMP2}^{decay}$, and $k_{Ligand}^{decay}$ are decay coefficients of VEGF (8.2×10^−6^ s^-1^), MMP-2 (0.0017 s^-1^), and ligands (0.0001 s^-1^), respectively. $k_{Ligand}^{deg}$ is a degradation coefficient of ECM (1.04×10^6^ M^-1^s^-1^). $k_{TIMP2:MMP2}^{on}$ is a kinetic association rate constant for binding TIMP-2 with MMP2 (5×10^5^ M^-1^s^-1^) and its term physically represents the reduction of MMP-2 by the endogenous soluble inhibitor TIMP-2. $k_{Complex:MT1-MMP}^{on}$ is a kinetic association rate constant for binding the ternary complex with MT1-MMP (1.95×10^4^M^-1^s^-1^), which results in the release of activated MMP-2. $k_{TIMP2:MT1-MMP}^{on}$ is a kinetic association rate constant for binding TIMP-2 with MT1-MMP (2.74×10^6^M^-1^s^-1^), and $k_{Complex}^{off}$ is a kinetic dissociation rate constant of the ternary complex for unbinding TIMP2 and MT1-MMP (2×10^−4^ s^-1^). $\alpha_{TIMP2}\left(x_{base}^{f}\right)$ and $\alpha_{MT1-MMP}\left(x_{base}^{f}\right)$ represent secretion rates of TIMP2 (1.0×10^-3^M s^-1^) and MT1-MMP (1.0×10^-1^M s^-1^) at the root of a filopodium, respectively.

### Numerical methods of “cell migration model in 3D ECM”

Cell migration simulations were carried out using a fourth order Rosenbrock method based on an adaptive time-stepping technique for integrating ordinary differential equations with the convergence criterion <10^−4^. The ordinary differential equations of cell and filopodia models were numerically coupled to solve for unknown variables associated with the mesh node position vectors for both cell membrane, nucleus membrane, transduce layer, and filopodial membrane (see Table 1). In particular, cell membrane and transduce layer were coupled with the viscoelastic actin cortex using kelvin-voigt model (Fig 1B). To solve two coupled ordinary differential equations in the **CC** and **CT** modules (Table 1) numerically, these equations should be converted with respect to vectors ${\left\{\frac{dx_{i}^{c}}{dt},\frac{dx_{i}^{t}}{dt}\right\}}^{T}$ as followings:

$$\left(\begin{array}{c} \frac{dx_{i}^{c}}{dt}\\ \frac{dx_{i}^{t}}{dt} \end{array}\right)=\frac{1}{C_{c}C_{t}+C_{cort}\left(C_{c}+C_{t}\right)}\left(\begin{array}{cc} C_{t}+C_{cort} & C_{cort}\\ C_{cort} & C_{c}+C_{cort} \end{array}\right)\left(\begin{array}{c} F_{FA,i}^{c}+F_{E,i}^{c}+F_{L,i}^{c}+F_{T,i}^{c}\\ F_{E,i}^{t}+F_{SF,i}^{t}+F_{T,i}^{t} \end{array}\right)\;i=1,\cdots ,N_{c}.$$ (10)

For cell migration simulation the Rosenbrock method outperforms the standard Runge—Kutta method which requires a relatively large number of iterations. Furthermore, the Rosenbrock method consumes less computing time by using adaptive time-step control that ranges from 10^−3^ s to 10^−2^ s in the present work. Thus, it is suitable for simulating transient cell migratory behaviours over 1 hour. For computations of ECM fiber networks, numbers of ECM fiber nodes were ranged from 30,000 to 234,000 depending on pore sizes of ECM model. As pore sizes of ECM fiber network models are smaller, computations of these models become more expensive. To solve ECM fiber network models effectively, computational domains of ECM fiber models were set within a radius of 15 μm at both the centre of cellular membrane and filopodial tip. As results, three ECM fiber network models (see Fig 4A, 4B and 4C) were visualized as half spheres. These computational domains were updated every 10 seconds of physical time as the cell interacts with ECM fibers dynamically.

One practical issue in computing finite mesh geometric models is to check geometrical compatibility. As the coordinates of cell membrane, filopodial membrane, and ECM fibril nodes are updated based on the equations of motion, geometrically incompatible situations occur occasionally in the configurations of the cell membrane mesh and that of the filopodia in relation to the ECM fibril surface. For example, some cell membrane nodes intersect with the fibril fiber, and the filopodia also intersects with the fibril fiber. These incompatible situations must be checked in every computational cycle, and necessary corrections such as contact forces (elastic repulsive) must be made [26,57].

### Microfluidic experiments for filopodia dynamics

The methods for microfluidic sprouting experiments were previously described in detail [58]. Briefly, GFP-expressing HUVEC (Angio-Proteomie) were cultured in EGM2-MV growth medium (Lonza) and used in experiments at passage 6. PDMS microfluidic devices were bonded to glass coverslips and channels were coated with PDL (Sigma). The central gel region was filled with 2.5 mg/ml Collagen I solution before incubating for 30 min at 37°C to polymerize. The NaOH concentration of the Collagen I solution was varied to control the pH of the solution during polymerization. After polymerization of the gel, HUVECs were seeded in the medium channel by introducing 40 μl of cell suspension at 2×10^6^ cells/ml. After 24 h, medium was replaced with EGM2-MV supplemented with 50 ng/ml VEGF (peprotech) and 250 nM S1P (Sigma). After 12 h, individual cells sprouting into the collagen gel were imaged at 60x magnification using a confocal laser-scanning microscope (FV-1000, Olympus). Collagen fibers were simultaneously visualized on the same instrument by collecting the reflected light (confocal reflectance microscopy). During imaging, cells were kept humidified at 37°C and 5% CO_2_. For imaging analysis, speeds of both filopodial tip and root was measured using a 3D image analysis tool (IMARIS, Bitplane).

## Supporting Information

S1 Fig Observation of oscillatory "load-and-fail" dynamics. A) experimental observations of oscillatory “load-and-fail” phenomena during the filopodial retractile phase over 1000 seconds; yellow arrows in ‘t+500s’ and ‘t+720s’ indicate directions of filopodial retractile movements, but blue arrow head in ‘t+740s’ represents the load (out-growth) of filopodial tip. 0.2 μm diameter fluorescent beads were embedded into collagen gel. An inset in each time point indicates an image of filopodial tip using RFP transfected HUVECs. Scale bars indicate 3 μm. B) the speed at the filopodial tip during the retractile phase over 1000 seconds. Oscillatory variation of speed represents ‘load-and-fail’ phenomena during the retractile phase.
(TIF)

S2 Fig A) Flow between the filopodia states (S) in filopodia penetration dynamical model which consists of ‘inactive phase(S = 0)’, ‘active phase (S = 1)’, ‘outgrowing phase (S = 2)’, ‘tugging phase (S = 3)’, ‘contractile phase (S = 4)’, ‘retractile phase (S = 5)’ and ‘decay phase (S = 6)’. Here, four major phases of S = 2, 3, 4 and 5 are highlighted as yellow colors. B) detailed algorithms of filopodia penetration dynamical model showing branched conditions for changing or keeping the filopodia states at next time t+Δt. Here, *L*_*f*_ is the length of filopodia, *L*_max_ is the maximum length of outgrowing filopodia (4.5 μm), and *L*_min_ is the minimum length of retractile filopodia (2 μm). *T*_*filo*_ is the elapsed time of filopodia penetration dynamics. The rupture test indicates the procedure to determine the completed breakage of bonds at the filopodial tip by the Bell’s equation. The polarity angle means the angle between direction of polarization for the cell and the unit vector normal to the local membrane where filopodial root is located. *N*_*filo*_ represents a unit normal vector parallel to the direction of the filopodial outgrowth and *N*_*fibr*_ represents a unit normal vector parallel to the orientation of neighboring ECM fiber at the filopodial tip.
(TIF)

S3 Fig Characterizations of three ECM fiber network models with pore sizes of 0.5, 1.0, and 1.5 μm. A) Bar graphs showing simulated moduli for three different single fiber diameters of 28, 34 and 41 nm for each ECM fiber network model. Note that identical single fiber modulus of 1 MPa is used for each ECM model. B) Bar graphs showing simulated moduli for four different conditions for each ECM model. Note that three different single fiber diameters of 28, 34 and 41 nm are used for three ECM models of 0.5, 1.0 and 1.5 μm, respectively. C) Liner regression (R^2^ = 0.898) of simulated moduli of ECM models (* marked cases in A) and B)) and experimentally measured relaxation moduli of collagen gel. Selected examples of deformed ECM network models of D) 0.5 μm, E) 1.0 μm and F) 1.5 μm at four different strains of 0, 0.3, 0.5 and 0.7, respectively.
(TIF)

S4 Fig ECM stretching model 1 with a pore size of 0.5μm. A) variations of stress under different stretching speeds of 0.25, 0.5. 0.75, and 1 nm/s and with identical single fiber diameter and Young’s modulus of 28 nm and 1 MPa, respectively. B) variations of stress under different single fiber’s Young’s moduli of 0.25, 0.5, 0.75, and 1 MPa and identical single fiber diameter of 28 nm and stretching speed of 0.5 nm/s, respectively. C) variations of stress under different single fiber’s diameters of 41, 34 and 28 nm and with identical single fiber’s Young’s modulus of 1 MPa and stretching speed of 0.5 nm/s. Red lines in each graph indicate linear fitting lines at the range of strain from 0.2 to 0.7. Here, slopes of red lines represent bulk modulus of ECM stretching model.
(TIF)

S5 Fig ECM stretching model 2 with a pore size of 1.0 μm. A) variations of stress under different stretching speeds of 0.25, 0.5, 0.75, and 1 nm/s and with identical single fiber diameter and Young’s modulus of 34 nm and 1 MPa, respectively. B) variations of stress under different single fiber’s Young’s moduli of 0.25, 0.5, 1, and 2 MPa and identical single fiber diameter of 34 nm and stretching speed of 0.5 nm/s, respectively. C) variations of stress under different single fiber’s diameters of 28, 34, and 41nm and with identical single fiber’s Young’s modulus of 1 MPa and stretching speed of 0.5 nm/s. Red lines in each graph indicate linear fitting lines at the range of strain from 0.2 to 0.7. Here, slopes of red lines represent bulk modulus of ECM stretching model.
(TIF)

S6 Fig ECM stretching model 3 with a pore size of 1.5 μm. A) variations of stress under different stretching speeds of 0.25, 0.5, 0.75, and 1 nm/s and with identical single fiber diameter and Young’s modulus of 41 nm and 1 MPa, respectively. B) variations of stress under different single fiber’s Young’s moduli of 0.25, 0.5, 1, and 2 MPa and identical single fiber diameter of 41 nm and stretching speed of 0.5 nm/s, respectively. C) variations of stress under different single fiber’s diameters of 28, 34, 41 nm and with identical single fiber’s Young’s modulus of 1 MPa and stretching speed of 0.5 nm/s. Red lines in each graph indicate linear fitting lines at the range of strain from 0.2 to 0.7. Here, slopes of red lines represent bulk modulus of ECM stretching model.
(TIF)

S7 Fig Contour plots of VEGF concentration at eight time points of 600, 1200, 1800, 2400, 3000, 3600, 4200, and 4800 seconds. Green bodies represent cellular and filopodial membranes. Blue body indicates nuclear membrane. Red lines indicate actin stress fibers. Scale bar is 5 μm.
(TIF)

S8 Fig Contour plots of MMP-2 concentration at eight time points of 600, 1200, 1800, 2400, 3000, 3600, 4200, and 4800 seconds. Green bodies represent cellular and filopodial membranes. Blue body indicates nuclear membrane. Red lines indicate actin stress fibers. Scale bar is 5 μm.
(TIF)

S9 Fig Contour plots of TIMP-2 concentration at eight time points of 600, 1200, 1800, 2400, 3000, 3600, 4200, and 4800 seconds. Green bodies represent cellular and filopodial membranes. Blue body indicates nuclear membrane. Red lines indicate actin stress fibers. Scale bar is 5 μm.
(TIF)

S10 Fig Deep cell invasion into a ECM fiber network model with pore sizes of 1.5 μm at eight time points of 600, 1200, 1800, 2400, 3000, 3600, 4200, and 4800 seconds. Black lines indicate collagen fibers with a diameter of 42 nm. Green bodies represent cellular and filopodial membranes. Blue body indicates nuclear membrane. Red lines indicate actin stress fibers. Scale bar is 5 μm.
(TIF)

S11 Fig Construction of ECM fiber network model. A) Tetrahedral meshes with crosslink nodes (red spheres), B) segmented ECM fibers were generated between crosslink nodes. Yellow spheres indicate segmented ECM fiber nodes. C) A magnified view in blue circle mark in B) showing examples of three fibers’ connectivity with a crosslink node. Blue lines indicated crosslinks between an ECM fiber node and a crosslink node.
(TIF)

S12 Fig Traction simulation of ECM fiber network model with pore size of 1.5 μm. Selected examples of sectional contours and vector plots of ECM fiber speeds at different time points of 110, and 550 seconds, respectively.
(TIF)

S1 Text Simulations of stretching ECM fiber network models. (DOCX)

S2 Text Simulations of reaction-diffusion mass transfer in 3D ECM. (DOCX)

S1 Video Filopodia retractile phase. Experimental observations of oscillatory “load-and-fail” phenomena during the filopodia retractile phase over 1000 seconds.
(AVI)

S2 Video Filopodia tugging phase. Experimental observation shows that the binding sites of FCs advance along ECM fibers and that the bound ECM fibers are pushed or pulled by the filopodial tip. Green color indicates filopodial tip and shaft, and while lines indicate collagen fibers. This movie was processed using single sliced images. For full collapsed images of filopodia tugging phase, see S12 Video.
(WMV)

S3 Video Simulation of filopodia tugging phase. Example of a simulated filopodium penetration into ECM network model with pore size of 1.5 μm over 970 seconds. Cell and filopodial membranes are visualized with green. Blue lines represent ECM fibers with single fiber’s diameter of 41nm, and single fiber’s modulus of 1 MPa. Scale bar is 500 nm. It shows that the filopodial tip crawls along ECM fibers, tugs neighbouring fibers, and contracts depending on the binding strength and stiffness and pore size of the ECM.
(WMV)

S4 Video Simulation of stretching ECM fiber network model. An example of the simulation of stretching ECM fiber network model with pore size of 0.5 μm, single fiber’s diameter of 28 nm, and single fiber’s modulus of 1 MPa. Constant stretching speed of 0.5 nm/s was imposed at both left and right sides of the ECM fiber model.
(AVI)

S5 Video Simulation of cell invasion into an ECM fiber network model 1. An example of simulated cell invasion into ECM fiber network model with pore sizes of 0.5 μm over 1300 seconds. Cell and filopodial membranes are visualized with green. Black lines represent ECM fibers with single fiber’s diameter of 28 nm, and single fiber’s modulus of 1 MPa. Scale bar is 5 μm.
(AVI)

S6 Video Simulation of cell invasion into an ECM fiber network model 2. An example of simulated cell invasion into ECM fiber network model with pore sizes of 1.0 μm over 1300 seconds. Cell and filopodial membranes are visualized with green. Black lines represent ECM fibers with single fiber’s diameter of 34 nm, and single fiber’s modulus of 1 MPa. Scale bar is 5 μm.
(AVI)

S7 Video Simulation of cell invasion into an ECM fiber network model 3. An example of simulated cell invasion into ECM fiber network model with pore sizes of 1.5 μm over 1200 seconds. Cell and filopodial membranes are visualized with green. Black lines represent ECM fibers with single fiber’s diameter of 41 nm, and single fiber’s modulus of 1 MPa. Scale bar is 5 μm.
(AVI)

S8 Video Experimental observation of cell-ECM interactions in collagen gel with pH level of 9. This video shows substantial deformation and remodelling of collagen gel by filopodia-ECM interactions in collagen gel with pH level of 9 over 30 minutes. Time-interval of the video is 5 minutes.
(AVI)

S9 Video Experimental observation of cell-ECM interactions in collagen gel with pH level of 7. This video shows substantial deformation and remodelling of collagen gel by filopodia-ECM interactions in collagen gel with pH level of 7 over 60 minutes. Time-interval of the video is 5 minutes.
(AVI)

S10 Video Experimental observation of cell-ECM interactions in collagen gel with pH level of 5. This video shows substantial deformation and remodelling of collagen gel by filopodia-ECM interactions in collagen gel with pH level of 5 over 60 minutes. Time-interval of the video is 5 minutes.
(AVI)

S11 Video Experimental observation of collagen gel deformation and remodeling. It shows substantial deformation and remodelling of collagen gel by protrusive and contractile motions of filopodia over 60 minutes. Time-interval of the video is 2 minutes.
(AVI)

S12 Video Experimental observation of filopodial state changes. It shows filopodial state changes from tugging/contractile phases to the retractile phase. Background fiber images were eliminated from the original 3D confocal images. ECM fiber is marked with a blue sphere.
(AVI)

S13 Video Experimental observation of traction stress generation during filopodia tugging and contractile phases. This video shows the generation of traction fields in collagen gel as beads move towards the extending filopodium tip during the tugging and contractile phases. 200 nm-sized fluorescent beads are embedded into the collagen gel.
(MP4)

S14 Video Experimental observation of traction stress relaxation during the filopodia retractile phase. This video shows the relaxation of traction fields in collagen gel as beads move away from the filopodium during the retractile phase. 200 nm-sized fluorescent beads are embedded into the collagen gel.
(MP4)

S15 Video Simulation of traction distributions of ECM fiber network model. It shows sectional contours and vector plots of displacement in ECM fiber network model with pore size of 1.5 μm over 1000 seconds. The plot in the right and top shows the formation of interface between the cell and ECM fibers, and the dynamic graph in the right and bottom shows variations of numbers of integrin-collagen bonds at five filopodial tips, which represents the stochastic model.
(WMV)

S16 Video Simulation of cell invasion model with no degradation of the ECM fiber network. Example of simulated cell invasion into ECM fiber network model with pore sizes of 1.5 μm over 510 seconds. Cell and filopodial membranes are visualized with green. Blue lines represent ECM fibers with single fiber’s diameter of 41 nm, and single fiber’s modulus of 1 MPa. MT1-MMP deficient cell model. The right panel shows time-varying contour plots of MMP-2, TIMP-2, and VEGF in both ECM and fluid domains, respectively.
(AVI)

S17 Video Simulation of deep cell invasion model incorporating the degradation of the ECM fiber network. Example of simulated deep cell invasion into ECM fiber network model with pore sizes of 1.5 μm over 3200 seconds. Cell and filopodial membranes are visualized with green. Black lines represent ECM fibers with single fiber's diameter of 41 nm, and single fiber's modulus of 1 MPa.
(WMV)

S18 Video Degradability of ECM fiber network model. An example of the degradability of ECM fiber network model due to the secretion of MMP-2 at the root of filopodium. Cell and filopodial membranes are visualized with green. Black lines represent ECM fibers with single fiber’s diameter of 41nm, and single fiber’s modulus of 1 MPa. Yellow dots represent crosslink nodes. Two blue arrows indicate clusters of crosslink nodes which are decomposed into multiple elements of single fibers.
(WMV)

S19 Video Mechanical interactions between filopodia and ECM fibers. An example of gel compaction and fiber remodelling by filopodia penetration dynamics in ECM fiber network model with a pore size of 0.5 μm. Two yellow circles indicate significant gel compaction and fiber remodelling, and red arrows indicated directions of gel compaction toward to the cell.
(WMV)
