# Supplementary figures and images for: Cell Invasion Dynamics into a Three Dimensional Extracellular Matrix Fibre Network
Source: PLoS Comput Biol. 2015 Oct 5;11(10):e1004535. doi: 10.1371/journal.pcbi.1004535 (PMC4593642; doi:10.1371/journal.pcbi.1004535)

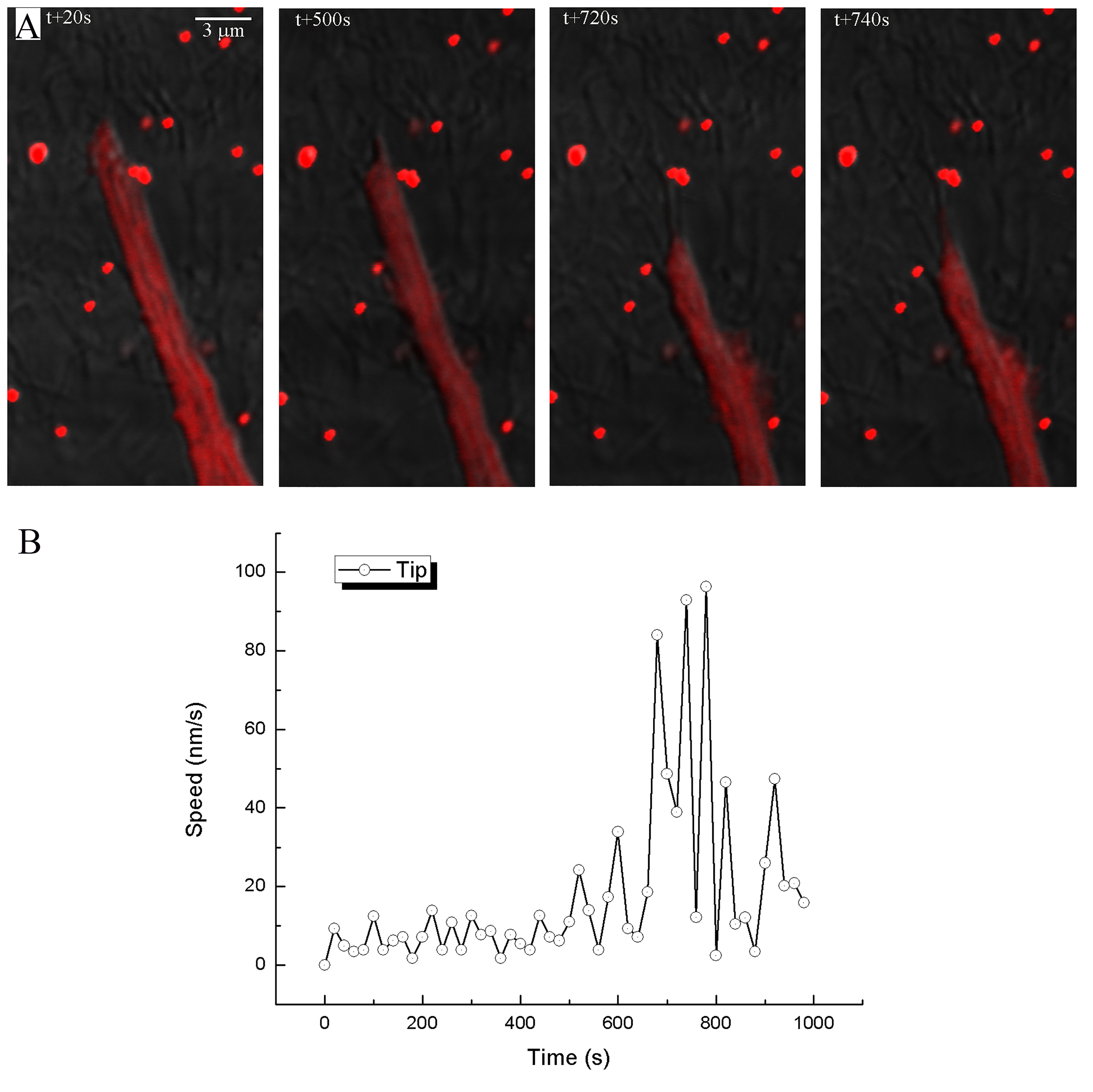

Supplement: S1 Fig — A) experimental observations of oscillatory “load-and-fail” phenomena during the filopodial retractile phase over 1000 seconds; yellow arrows in ‘t+500s’ and ‘t+720s’ indicate directions of filopodial retractile movements, but blue arrow head in ‘t+740s’ represents the load (out-growth) of filopodial tip. 0.2 μm diameter fluorescent beads were embedded into collagen gel. An inset in each time point indicates an image of filopodial tip using RFP transfected HUVECs. Scale bars indicate 3 μm. B) the speed at the filopodial tip during the retractile phase over 1000 seconds. Oscillatory variation of speed represents ‘load-and-fail’ phenomena during the retractile phase. (TIF) [file pcbi.1004535.s001.tif]

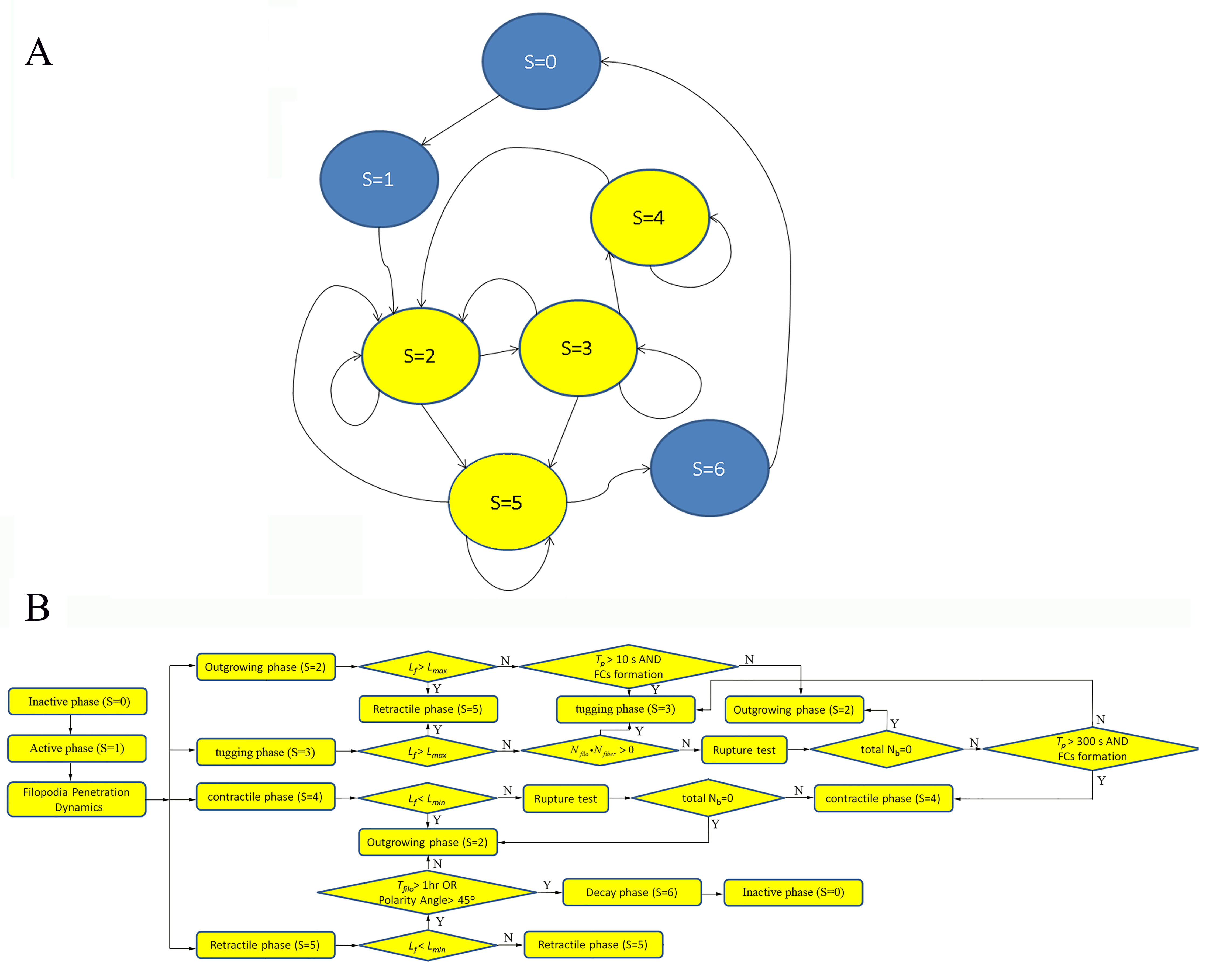

Supplement: S2 Fig — Here, four major phases of S = 2, 3, 4 and 5 are highlighted as yellow colors. B) detailed algorithms of filopodia penetration dynamical model showing branched conditions for changing or keeping the filopodia states at next time t+Δt. Here, L f is the length of filopodia, L max is the maximum length of outgrowing filopodia (4.5 μm), and L min is the minimum length of retractile filopodia (2 μm). T filo is the elapsed time of filopodia penetration dynamics. The rupture test indicates the procedure to determine the completed breakage of bonds at the filopodial tip by the Bell’s equation. The polarity angle means the angle between direction of polarization for the cell and the unit vector normal to the local membrane where filopodial root is located. N filo represents a unit normal vector parallel to the direction of the filopodial outgrowth and N fibr represents a unit normal vector parallel to the orientation of neighboring ECM fiber at the filopodial tip. (TIF) [file pcbi.1004535.s002.tif]

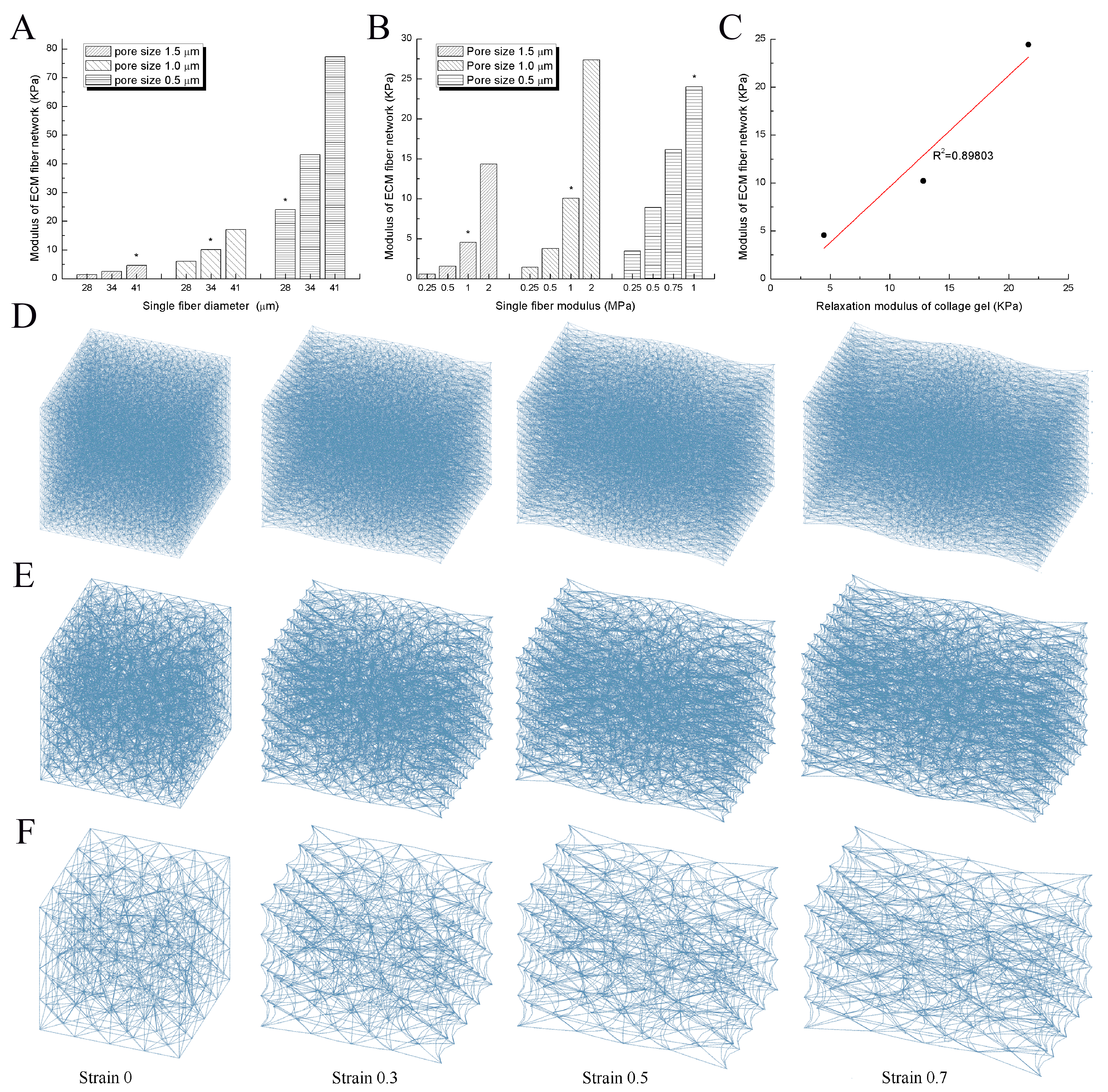

Supplement: S3 Fig — A) Bar graphs showing simulated moduli for three different single fiber diameters of 28, 34 and 41 nm for each ECM fiber network model. Note that identical single fiber modulus of 1 MPa is used for each ECM model. B) Bar graphs showing simulated moduli for four different conditions for each ECM model. Note that three different single fiber diameters of 28, 34 and 41 nm are used for three ECM models of 0.5, 1.0 and 1.5 μm, respectively. C) Liner regression (R2 = 0.898) of simulated moduli of ECM models (* marked cases in A) and B)) and experimentally measured relaxation moduli of collagen gel. Selected examples of deformed ECM network models of D) 0.5 μm, E) 1.0 μm and F) 1.5 μm at four different strains of 0, 0.3, 0.5 and 0.7, respectively. (TIF) [file pcbi.1004535.s003.tif]

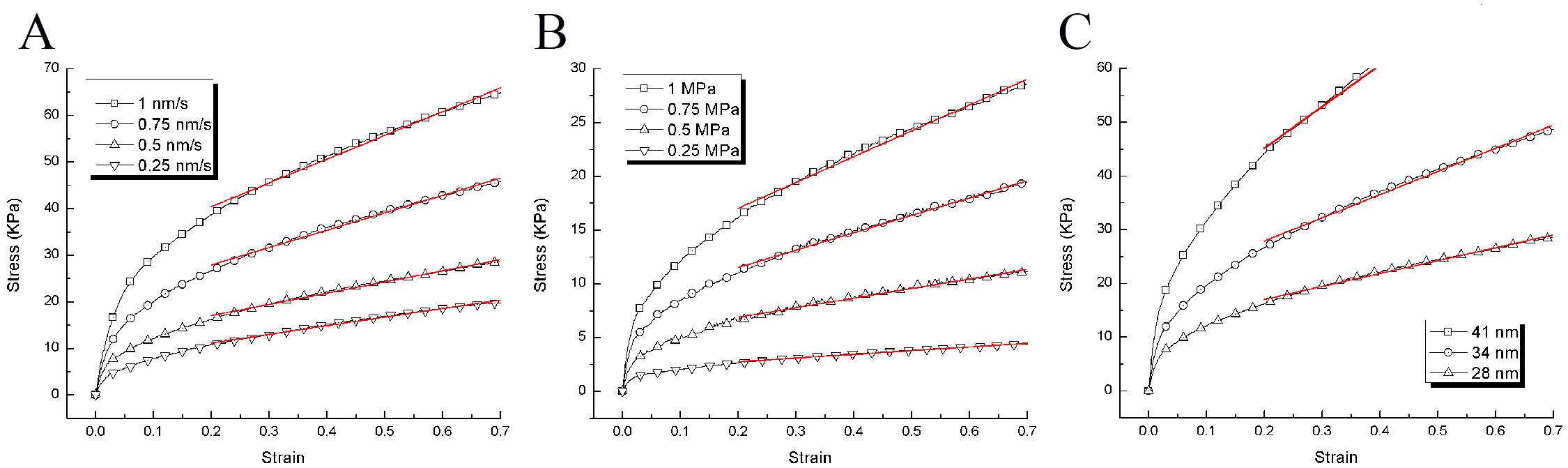

Supplement: S4 Fig — A) variations of stress under different stretching speeds of 0.25, 0.5. 0.75, and 1 nm/s and with identical single fiber diameter and Young’s modulus of 28 nm and 1 MPa, respectively. B) variations of stress under different single fiber’s Young’s moduli of 0.25, 0.5, 0.75, and 1 MPa and identical single fiber diameter of 28 nm and stretching speed of 0.5 nm/s, respectively. C) variations of stress under different single fiber’s diameters of 41, 34 and 28 nm and with identical single fiber’s Young’s modulus of 1 MPa and stretching speed of 0.5 nm/s. Red lines in each graph indicate linear fitting lines at the range of strain from 0.2 to 0.7. Here, slopes of red lines represent bulk modulus of ECM stretching model. (TIF) [file pcbi.1004535.s004.tif]

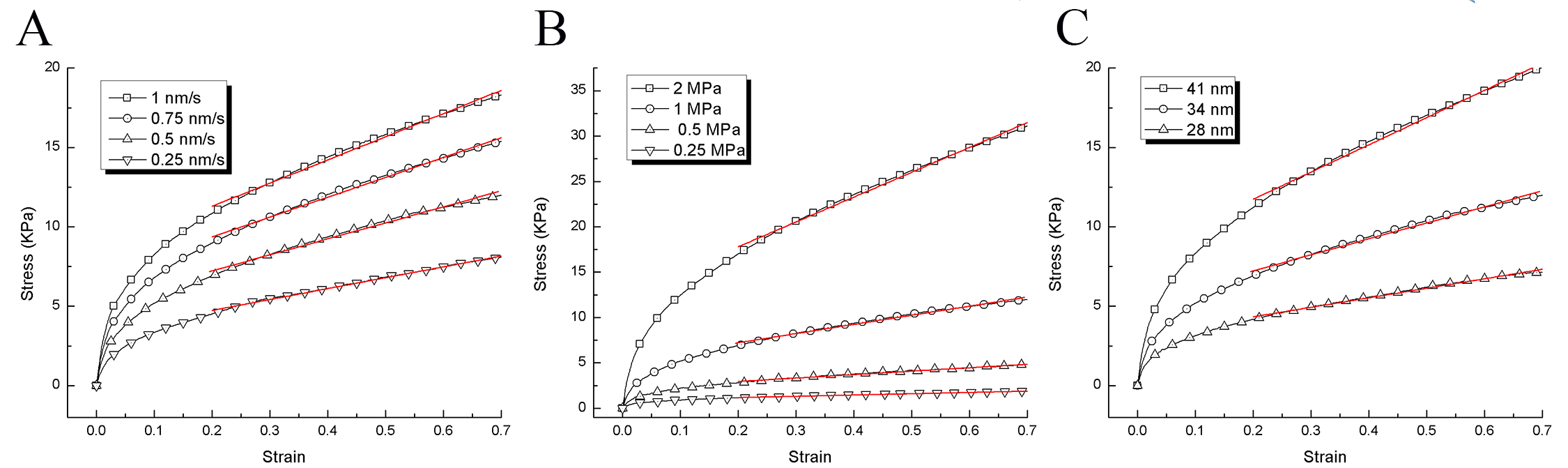

Supplement: S5 Fig — A) variations of stress under different stretching speeds of 0.25, 0.5, 0.75, and 1 nm/s and with identical single fiber diameter and Young’s modulus of 34 nm and 1 MPa, respectively. B) variations of stress under different single fiber’s Young’s moduli of 0.25, 0.5, 1, and 2 MPa and identical single fiber diameter of 34 nm and stretching speed of 0.5 nm/s, respectively. C) variations of stress under different single fiber’s diameters of 28, 34, and 41nm and with identical single fiber’s Young’s modulus of 1 MPa and stretching speed of 0.5 nm/s. Red lines in each graph indicate linear fitting lines at the range of strain from 0.2 to 0.7. Here, slopes of red lines represent bulk modulus of ECM stretching model. (TIF) [file pcbi.1004535.s005.tif]

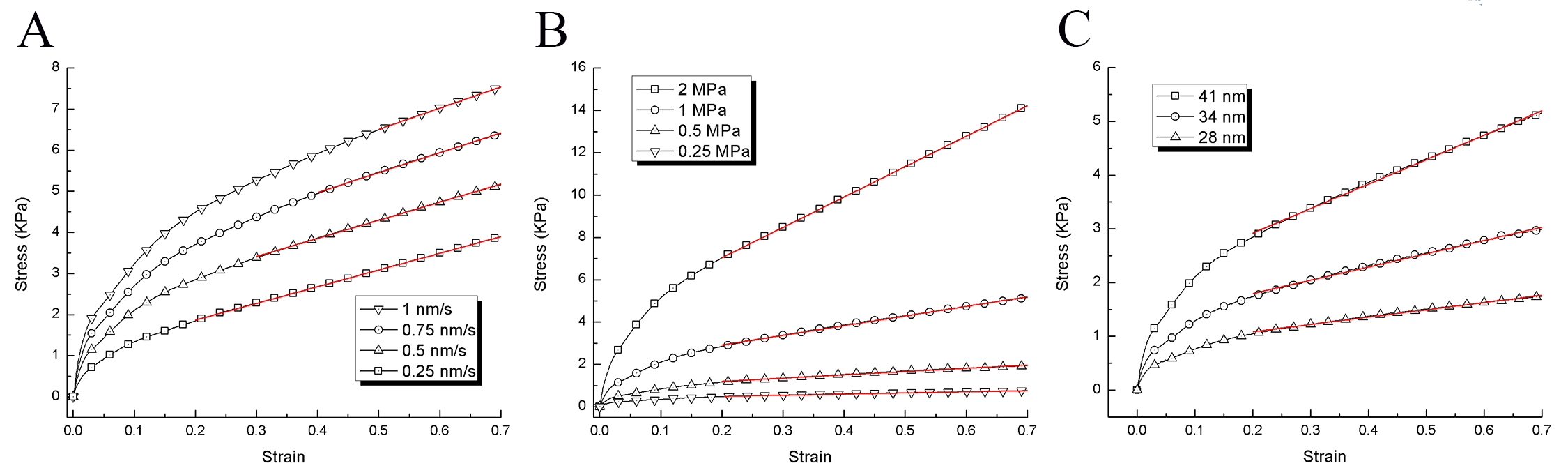

Supplement: S6 Fig — A) variations of stress under different stretching speeds of 0.25, 0.5, 0.75, and 1 nm/s and with identical single fiber diameter and Young’s modulus of 41 nm and 1 MPa, respectively. B) variations of stress under different single fiber’s Young’s moduli of 0.25, 0.5, 1, and 2 MPa and identical single fiber diameter of 41 nm and stretching speed of 0.5 nm/s, respectively. C) variations of stress under different single fiber’s diameters of 28, 34, 41 nm and with identical single fiber’s Young’s modulus of 1 MPa and stretching speed of 0.5 nm/s. Red lines in each graph indicate linear fitting lines at the range of strain from 0.2 to 0.7. Here, slopes of red lines represent bulk modulus of ECM stretching model. (TIF) [file pcbi.1004535.s006.tif]

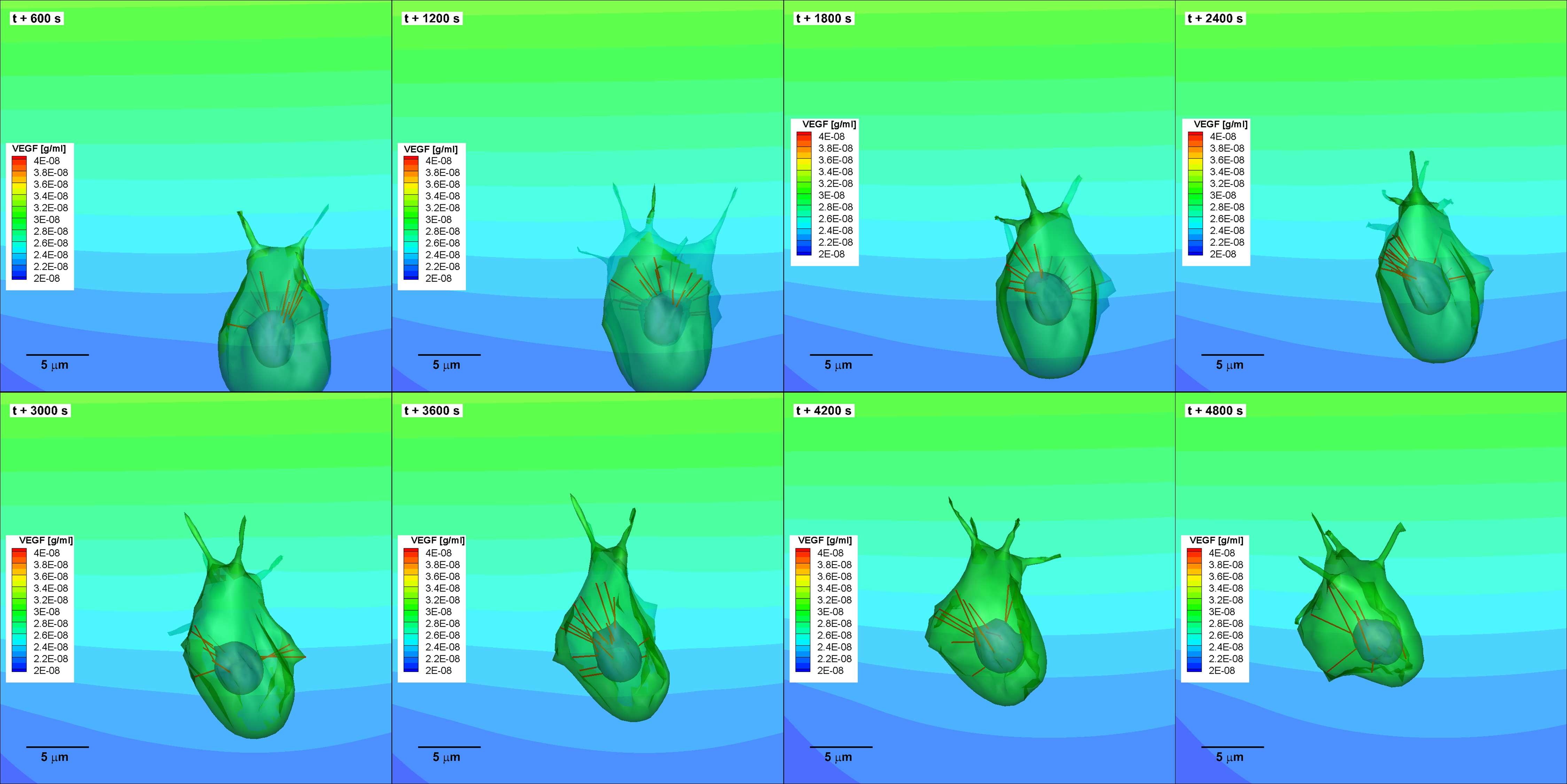

Supplement: S7 Fig — Green bodies represent cellular and filopodial membranes. Blue body indicates nuclear membrane. Red lines indicate actin stress fibers. Scale bar is 5 μm. (TIF) [file pcbi.1004535.s007.tif]

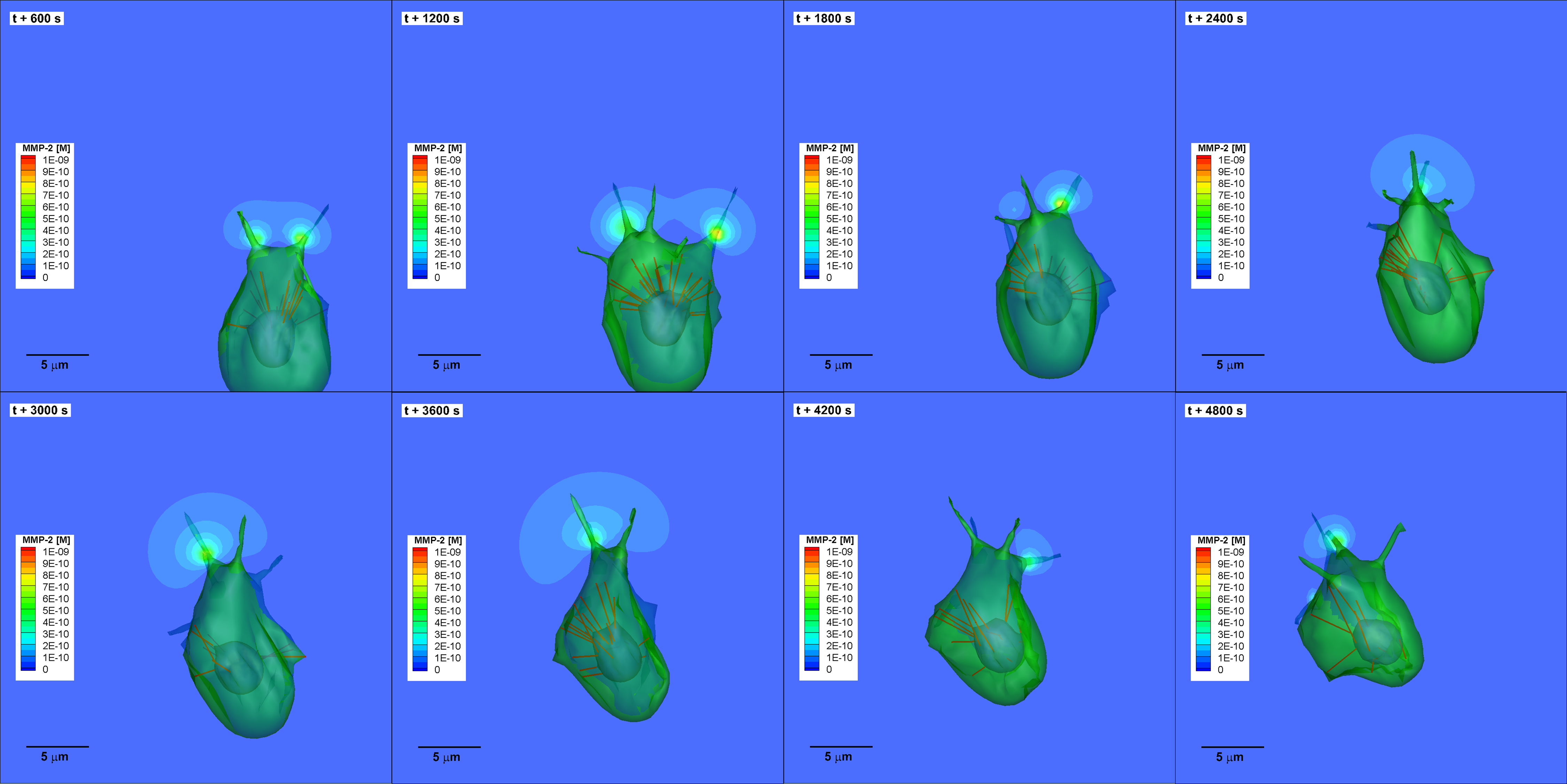

Supplement: S8 Fig — Green bodies represent cellular and filopodial membranes. Blue body indicates nuclear membrane. Red lines indicate actin stress fibers. Scale bar is 5 μm. (TIF) [file pcbi.1004535.s008.tif]

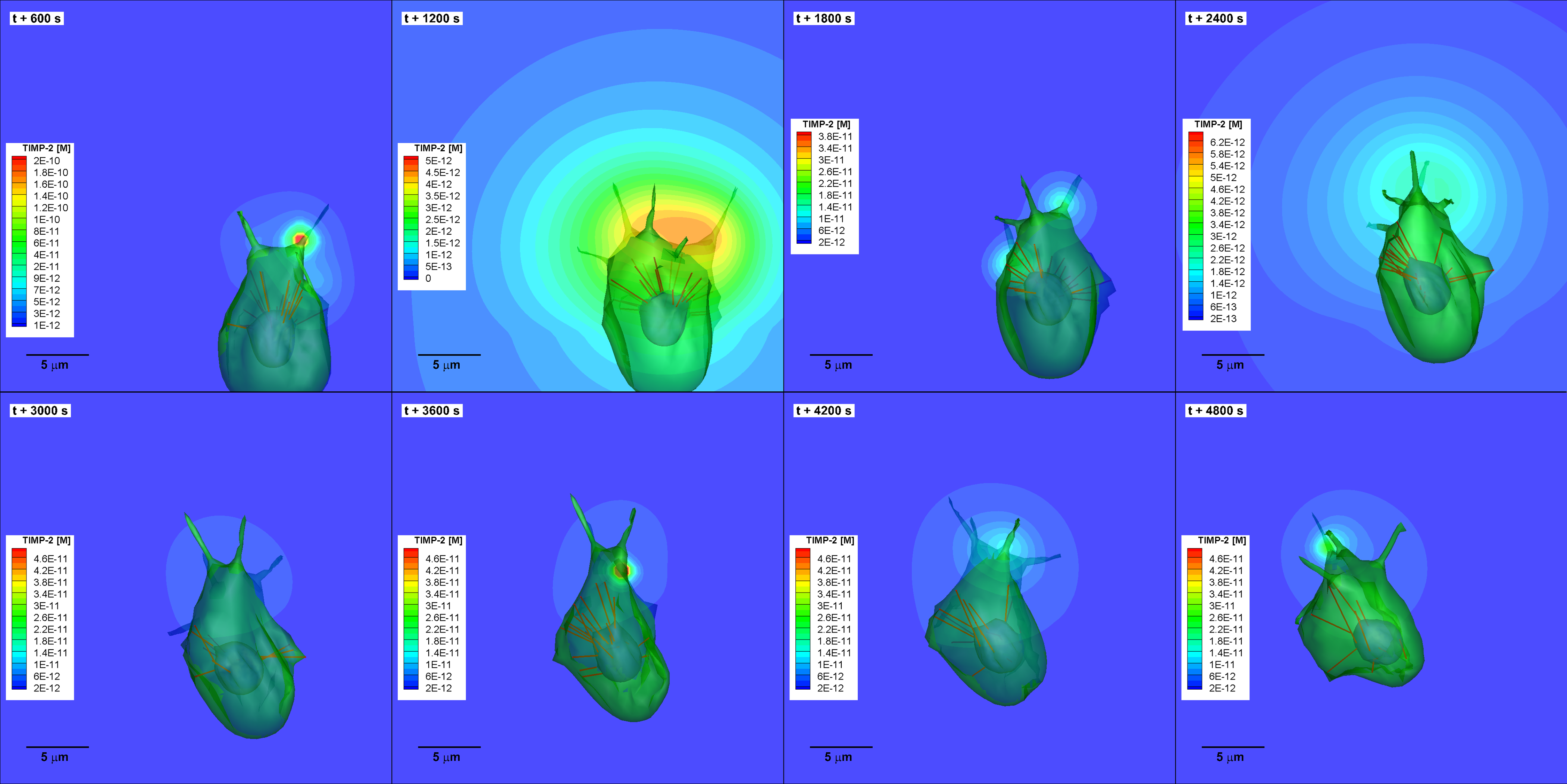

Supplement: S9 Fig — Green bodies represent cellular and filopodial membranes. Blue body indicates nuclear membrane. Red lines indicate actin stress fibers. Scale bar is 5 μm. (TIF) [file pcbi.1004535.s009.tif]

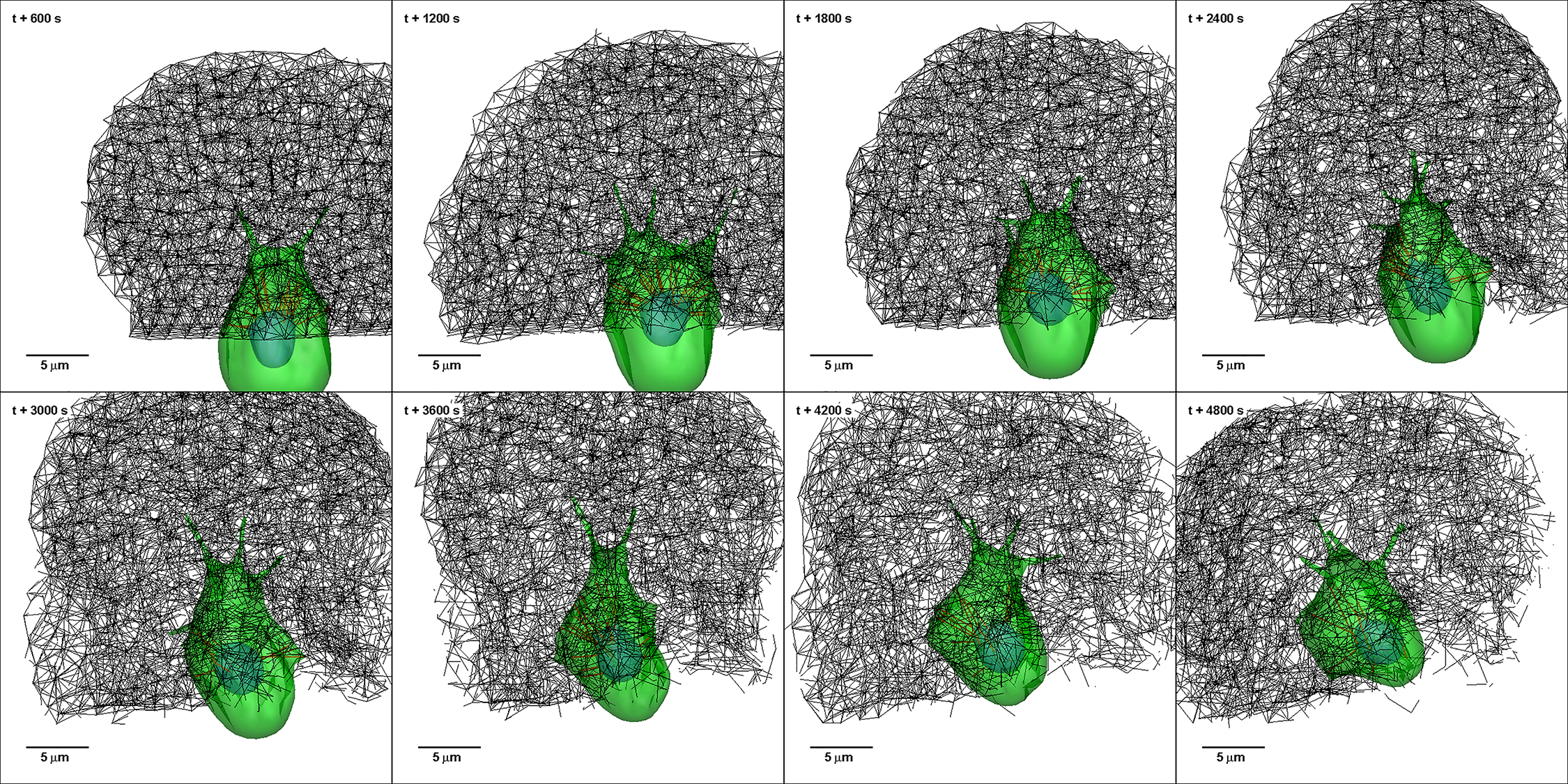

Supplement: S10 Fig — Black lines indicate collagen fibers with a diameter of 42 nm. Green bodies represent cellular and filopodial membranes. Blue body indicates nuclear membrane. Red lines indicate actin stress fibers. Scale bar is 5 μm. (TIF) [file pcbi.1004535.s010.tif]

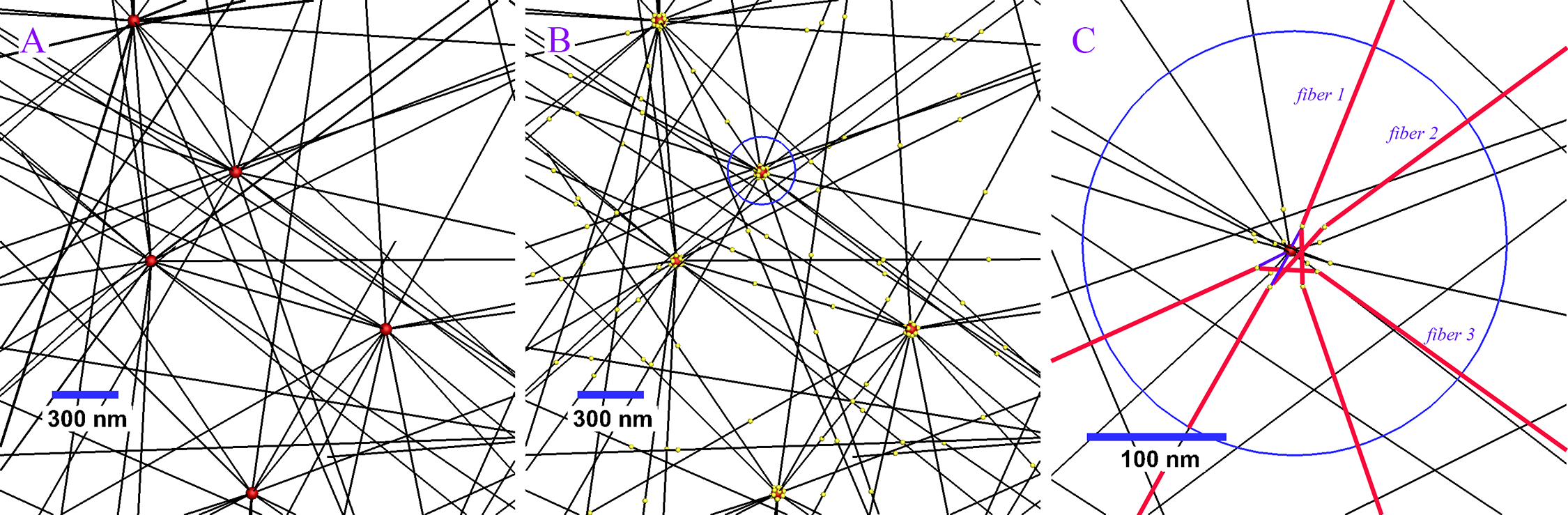

Supplement: S11 Fig — A) Tetrahedral meshes with crosslink nodes (red spheres), B) segmented ECM fibers were generated between crosslink nodes. Yellow spheres indicate segmented ECM fiber nodes. C) A magnified view in blue circle mark in B) showing examples of three fibers’ connectivity with a crosslink node. Blue lines indicated crosslinks between an ECM fiber node and a crosslink node. (TIF) [file pcbi.1004535.s011.tif]

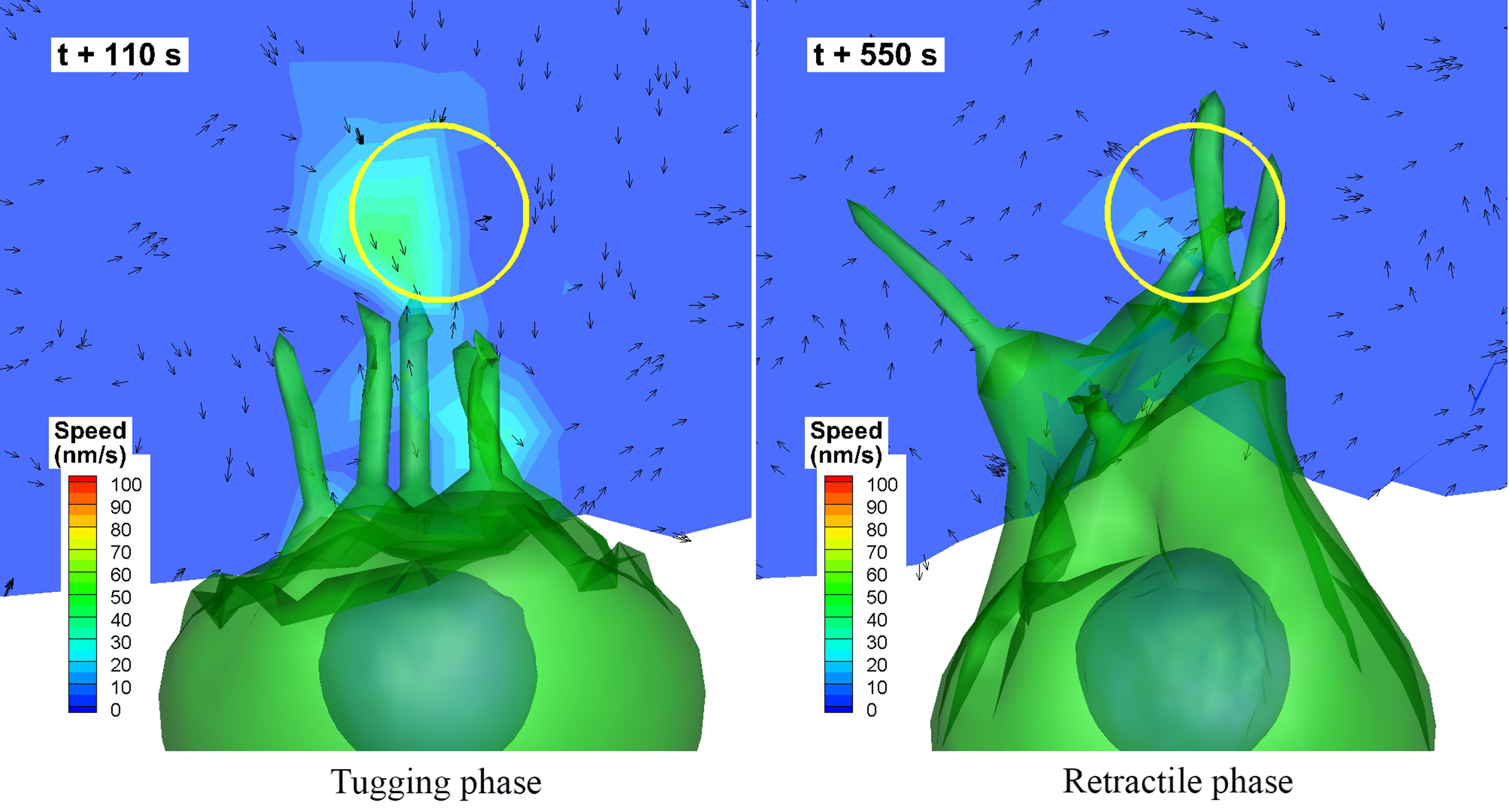

Supplement: S12 Fig — Selected examples of sectional contours and vector plots of ECM fiber speeds at different time points of 110, and 550 seconds, respectively. (TIF) [file pcbi.1004535.s012.tif]
