# Supplementary material for: Cell Invasion Dynamics into a Three Dimensional Extracellular Matrix Fibre Network
Source: PLoS Comput Biol. 2015 Oct 5;11(10):e1004535. doi: 10.1371/journal.pcbi.1004535 (PMC4593642; doi:10.1371/journal.pcbi.1004535)
Supplement: S1 Text — (DOCX) [file pcbi.1004535.s013.docx]

**Text S1**

***Simulations of stretching ECM fiber network models***

For the simulation of stretching ECM fiber network models, three ECM stretching models with pore sizes of 0.5, 1.0 and 1.5 μm, respectively, and fiber diameters of 28, 34 and 41 nm, respectively, were built with cube structures with equilateral length of 10 um as shown in Figure S3d, S3e and S3f. As for boundary conditions for the stretching simulations, constant stretching speed conditions were imposed at both left and right sides of each ECM model. After testing stretching simulation with various stretching speeds, we found that the stretching speed less than 0.5 nm/s at both sides of the ECM model gives the convergence in simulated values of bulk moduli (Figure S4a, S5a and S6a). Thereafter, this stretching speed was selected to simulate various cases of stretching ECM fiber network models under different single fiber moduli (Figure S4b, S5b and S6b) and single fiber diameters (Figure S4c, S5c and S6c).

As for ECM fiber network model 1 with pore sizes of 0.5 µm, the first stretch tests were simulated with four different stretching speeds of 0.25, 0.5, 0.75 and 1 nm/s, identical single fiber modulus of 1 MPa and identical single fiber diameter of 28 nm. Computationally measured bulk moduli under different stretching speeds of 0.25, 0.5, 0.75 and 1 nm/s are calculated as 18.43, 24.01, 37.29 and 50.98 KPa, respectively (Figure S4a). The second stretch tests were further simulated for ECM fiber network model 1 with four different single fiber’s moduli of 0.25, 0.5, 0.75 and 1 MPa, identical fiber diameter of 28 nm and identical stretching speed of 0.5 nm/s. As results, computationally measured bulk moduli for four different single fiber’s moduli of 0.25, 0.5, 0.75 and 1 MPa are calculated as 3.44, 8.90, 16.16 and 24.01 KPa, respectively (Figure S4b). Lastly, the third stretching tests were also simulated for ECM fiber network model 1 with three different single fiber’s diameters of 28, 34 and 41 nm, identical single fiber’s modulus of 1 MPa and identical stretching speed of 0.5 nm/s. As results, bulk moduli of the ECM stretching model 1 under different fiber’s diameters of 28, 34 and 41 nm are calculated as 24.01, 43.16 and 77.32 KPa, respectively (Figure S4c).

As for ECM fiber network model 2 with pore sizes of 1 µm, the first stretch tests were simulated with four different stretching speeds of 0.25, 0.5, 0.75 and 1 nm/s, identical single fiber modulus of 1 MPa and identical single fiber diameter of 34 nm. Computationally measured bulk moduli under different stretching speeds of 0.25, 0.5, 0.75 and 1 nm/s are calculated as 6.86, 10.07, 12.51 and 14.61 KPa, respectively (Figure S5a). The second stretch tests were further simulated for ECM fiber network model 2 with four different single fiber’s moduli of 0.25, 0.5, 1 and 2 MPa, identical fiber diameter of 34 nm and identical stretching speed of 0.5 nm/s. As results, computationally measured bulk moduli for four different single fiber’s moduli of 0.25, 0.5, 1 and 2 MPa are calculated as 1.41, 3.77, 10.07 and 27.38 KPa, respectively (Figure S5b). Lastly, the third stretching tests were also simulated for ECM fiber network model 2 with three different single fiber’s diameters of 28, 34 and 41 nm, identical single fiber’s modulus of 1 MPa and identical stretching speed of 0.5 nm/s. As results, bulk moduli of the ECM stretching model 2 under different fiber’s diameters of 28, 34 and 41 nm are calculated as 5.98, 10.07 and 17.14 KPa, respectively (Figure S5c).

As for ECM fiber network model 3 with pore sizes of 1.5 µm, the first stretch tests were simulated with four different stretching speeds of 0.25, 0.5, 0.75 and 1 nm/s, identical single fiber modulus of 1 MPa and identical single fiber diameter of 41 nm. Here, red lines in each graph indicate linear correlations calculated at the range of strain rates from 0.2 and to 0.7, and those slopes represent bulk moduli of the ECM model. Computationally measured bulk moduli under different stretching speeds of 0.25, 0.5, 0.75 and 1 nm/s are calculated as 4.06, 4.40, 4.84 and 5.18 KPa, respectively (Figure S6a). The second stretch tests were further simulated for ECM fiber network model 3 with four different single fiber’s moduli of 0.25, 0.5, 1 and 2 MPa, identical fiber diameter of 41 nm and identical stretching speed of 0.5 nm/s. As results, computationally measured bulk moduli for four different single fiber’s moduli of 0.25, 0.5, 1 and 2 MPa are calculated as 0.54, 1.54, 4.55 and 14.36 KPa, respectively (Figure S6b). Lastly, the third stretching tests were also simulated for ECM fiber network model 3 with three different single fiber’s diameters of 28, 34 and 41 nm, identical single fiber’s modulus of 1 MPa and identical stretching speed of 0.5 nm/s. As results, bulk moduli of the ECM stretching model 3 under different fiber’s diameters of 28, 34 and 41 nm are calculated as 1.36, 2.47 and 4.55 KPa, respectively (Figure S6c).
