# Supplementary material for: Cell Invasion Dynamics into a Three Dimensional Extracellular Matrix Fibre Network
Source: PLoS Comput Biol. 2015 Oct 5;11(10):e1004535. doi: 10.1371/journal.pcbi.1004535 (PMC4593642; doi:10.1371/journal.pcbi.1004535)
Supplement: S2 Text — (DOCX) [file pcbi.1004535.s014.docx]

**Text S2**

***Simulations of reaction-diffusion mass transfer in 3D ECM***

Four distinct mechanisms in 3D ECM associated with chemotaxis, proteolysis, haptotaxis, and degradation are modelled to incorporate chemical interactions of 3D ECM with filopodia and cellular membranes (Figure 1-C). As for the computation of concentrations of VEGF, MMP-2, and TIMP-2, computational domains consist of three components: 1) a ECM domain, into which each biochemical is secreted and diffused, 2) a sprout surface layer, and 3) a fluid domain. Three kinds of boundary conditions are imposed at computational domains. First, at the ECM domain, in case of VEGF concentration, constant boundary condition was imposed at the top surface of ECM domain as 40 ng/ml, and zero flux boundary conditions were imposed four lateral surfaces in the ECM domain ($\nabla C_{VEGF,ECM}=0$). However, in case of concentrations of MMP-2 and TIMP-2, zero flux boundary conditions were imposed for four lateral surfaces as well as one top surface in the ECM domain. Second, conjugate mass transfer condition was imposed at the sprout surface layer (or interface between ECM and fluid domains) ($D_{X,ECM}\nabla C_{X,ECM}=D_{X,fluid}\nabla C_{X,fluid}$) for concentrations of VEGF, MMP-2, and TIMP-2. Last, at the fluid domain, in case of VEGF concentration, constant boundary conditions were imposed at four lateral surfaces and one bottom surface in the fluid domain as 20 ng/ml. However, in case of concentrations of MMP-2 and TIMP-2, zero flux boundary conditions were imposed for four lateral surfaces as well as one bottom surface in the ECM domain.

Two-way couplings between VECF solver and cell mechanics solver including filopodia and cellular membrane mechanics were considered: 1) the direction of each outgrowing filopodium was determined by the gradient of VEGF concentration at the filopodial tip, and 2) sprout surface layer was updated as cellular membrane interact with ECM fibers, that is, the phase of computational domain is changed from ECM to fluid as the sprout surface layer grows. As simulated results shown in Figure S7, as the cell made a deep invasion into the ECM domain, filopodial tips were found to orient towards the top surface of ECM domain with a chemotactic cue of 40 ng/ml. In addition, contour lines are found to bend more at the sprout surface, as the cell invades into ECM domain deeper.

As for the computation of MMP-2 concentration, MMP-2 concentration influences the degradation of ECM integrity ($\frac{dC_{ECM}}{dt}=-k_{ECM}^{deg}C_{MMP2}C_{ECM}$), and the reduction of TIMP-2 concentration binding with MMP-2 concentration. Subsequently, the degradation of ECM integrity influences the disassembly of crosslinked ECM fiber network. Next, uncrosslinked ECM fibers are remodeled, and new sprout surface layer is extended as the cell mechanically interacts with ECM fibers. As simulated results shown in Figure S8, as the cell made a deep invasion into the ECM domain, MMP-2 is secreted at the root of filopodia. Highest concentration of MMP-2 is found at the root of filopodia, and its concentration decays at the concentric circles from the root of filopodia. It should be noted that rates of secreted MMP-2 are varied depending on binding MT1-MMP with the ternary complex.

As for the computation of TIMP-2 concentration, TIMP-2 concentration is decreased with increases in concentrations of MMP-2 and MT1-MMP. However, its concentration is increased due to kinetic dissociation of the ternary complex for unbinding TIMP-2 and MT1-MMP. As simulated results shown in Figure S9, as the cell made a deep invasion into the ECM domain, TIMP-2 concentrations are highest at time points of 600 and 3600 s (140 and 38 pM, respectively), which results in the inhibition of MMP-2 at identical time points (400 and 500 pM in Figure S9). However, when TIMP-2 concentration is lowest at the time point of 1200 s (4 pM), the secretion of MMP-2 is found to increase (800 pM in Figure S8).
